# Current Challenges and Future Perspectives of Renal Tubular Dysfunction in Diabetic Kidney Disease

**DOI:** 10.3389/fendo.2021.661185

**Published:** 2021-06-10

**Authors:** Suyan Duan, Fang Lu, Dandan Song, Chengning Zhang, Bo Zhang, Changying Xing, Yanggang Yuan

**Affiliations:** Department of Nephrology, The First Affiliated Hospital of Nanjing Medical University, Nanjing Medical University, Nanjing, China

**Keywords:** renal tubular dysfunction, tubular biomarkers, sodium-glucose cotransporter-2, diabetic kidney disease, therapeutic strategies

## Abstract

Over decades, substantial progress has been achieved in understanding the pathogenesis of proteinuria in diabetic kidney disease (DKD), biomarkers for DKD screening, diagnosis, and prognosis, as well as novel hypoglycemia agents in clinical trials, thereby rendering more attention focused on the role of renal tubules in DKD. Previous studies have demonstrated that morphological and functional changes in renal tubules are highly involved in the occurrence and development of DKD. Novel tubular biomarkers have shown some clinical importance. However, there are many challenges to transition into personalized diagnosis and guidance for individual therapy in clinical practice. Large-scale clinical trials suggested the clinical relevance of increased proximal reabsorption and hyperfiltration by sodium-glucose cotransporter-2 (SGLT2) to improve renal outcomes in patients with diabetes, further promoting the emergence of renal tubulocentric research. Therefore, this review summarized the recent progress in the pathophysiology associated with involved mechanisms of renal tubules, potential tubular biomarkers with clinical application, and renal tubular factors in DKD management. The mechanism of kidney protection and impressive results from clinical trials of SGLT2 inhibitors were summarized and discussed, offering a comprehensive update on therapeutic strategies targeting renal tubules.

## Introduction

Along with the disease spectrum that evolved around the world over the past 30 years, diabetic kidney disease (DKD) has become the leading cause of end-stage kidney disease (ESKD) at daunting rates in both developed and developing countries (1, 2). Due to the increased risk of morbidity and mortality of DKD, the number of DKD related studies rapidly increased over the past two decades, with more than 27,500 papers published from 2000 to 2017 (3). Growing evidence suggests the underlying pathogenesis of DKD involves the renal proximal tubular epithelial cell dysfunction in a high glucose environment, oxidative stress, inflammation, fibrosis, and apoptosis (4). In addition, a large number of tubular biomarkers for DKD screening, diagnosis, and prognosis are tightly associated with the prognosis of the kidney in DKD, providing evidence for potential shifting of the paradigm from glomerulocentric to tubulocentric theory (5). It has been repeatedly shown that compared with the glomerular lesions, the extent of tubulointerstitial lesions correlates well with renal function, and the associated biomarkers have been identified (6). Urinary tubular injury markers may increase in patients with diabetes even before the onset of microalbuminuria (7, 8). Plasma tubular markers, which may reflect inflammatory and fibrotic responses, oxidative stress, and capacity of reabsorption in DKD, were also reported to be associated with early renal function decline and DKD progression (9, 10). Moreover, the biomarkers of tubulointerstitial function and structural changes were ultimately proven to be better predictors of disease progression and long-term prognosis than the current markers (11). Current prognostic markers of DKD have certain limitations. Estimated glomerular filtration rate (eGFR) and albuminuria are only modestly useful for risk prediction in type 2 diabetes mellitus (T2DM) patients with preserved renal function, and DKD progresses even in the absence of albuminuria (12, 13). Most importantly, inhibition of proximal tubule glucose transport *via* sodium-glucose cotransporter-2 (SGLT-2) has shown nephroprotective effects in a variety of large-sample, multicenter, placebo-controlled, and randomized clinical trials. By investigating the mechanism of the newest disease-modifying treatments for DKD, an accumulating body of research had documented the vital role of tubule function in regulating glomerular filtration through tubuloglomerular feedback. Moreover, the growth of the proximal tubule in the diabetic context supplies muscular strength to the established status of renal tubules in DKD (14, 15). The tubuloglomerular feedback mechanism begins with the theory that diabetic hyperfiltration and glomerular capillary hypertension are significant treatable stressors contributing to the progression of DKD (16–19). In diabetic conditions, the increased filtered load of glucose results in an increase in sodium-coupled glucose reabsorption by the proximal tubule. Decreased sodium delivery to the macula densa subsequently inhibits adenosine-triphosphate (ATP) conversion into adenosine, which results in the vasodilation of the afferent arteriole and the intrarenal activation of the renin–angiotensin–aldosterone system (RAAS), ultimately inducing glomerular hypertension and kidney hyperfiltration (15, 16). Hence, hyperreabsorption of water and solutes has a central role in the regulation of eGFR, highlighting the importance of alteration in the tubuloglomerular feedback for the development of DKD.

This review aimed to summarize the latest updates on the pathogenesis of renal tubular dysfunction in DKD, potential applications of tubular biomarkers, and renal tubule-targeting therapeutics based on evidence from recent trials in DKD.

## New Insights Into the Pathophysiology of Renal Tubules in DKD

### Morphological Changes

Recently, there has been a growing consensus that tubular abnormalities, a consistent feature of DKD, are not the aftermath of glomerular damage. Tubular cells have the potential to be the primary targets for diverse pathophysiological influences (20). The shift has been suggested from the traditional paradigm of glomerulus-centered pathophysiology extended to the tubule-interstitium (21, 22). Morphological changes of tubulointerstitial lesions in DKD include thickening of the tubular basement membrane, tubular atrophy, interstitial inflammation, interstitial fibrosis, and vascular abnormalities (23). The association of tubulointerstitial lesions with DKD progression has been validated in several reports. A study in a Chinese population with an early stage of biopsy-proven DKD suggested that interstitial lesions and glomerular injuries were independently predictive of the time to ESKD (24). Another study from the United States population at relatively late stages of biopsy-proven DKD showed that interstitial fibrosis and tubular atrophy were of univariate significance for their ability to predict clinical prognosis (25). Moreover, the association of histological lesions with renal progression may differ in type 1 and type 2 DKD. In type 1 diabetes mellitus (T1DM), glomerular damage was observed through all stages. Nevertheless, minimal or no glomerular lesions but notable tubulointerstitial and/or arteriolar abnormalities were observed in type 2 DKD patients with microalbuminuria or overt proteinuria (26–28). Additionally, tubulointerstitial lesions were observed mainly in advanced disease and might contribute to the progression to ESKD in patients with T1DM (28, 29). The pathological disparity in different types of DM may be attributed to various other diabetogenic stimuli other than high glucose, including insulin resistance and growth factors and cytokines, which activate inflammatory, apoptosis, ischemic, pro-oxidant, and fibrotic pathways. A growing number of studies have proven that genes associated with pathological features of DKD are regulated not only by classical signaling pathways but also by epigenetic mechanisms involving chromatin histone modifications, deoxyribonucleic acid (DNA) methylation, and non-coding ribonucleic acid (RNA) (30).

### Functional Changes

**Figure 1:**
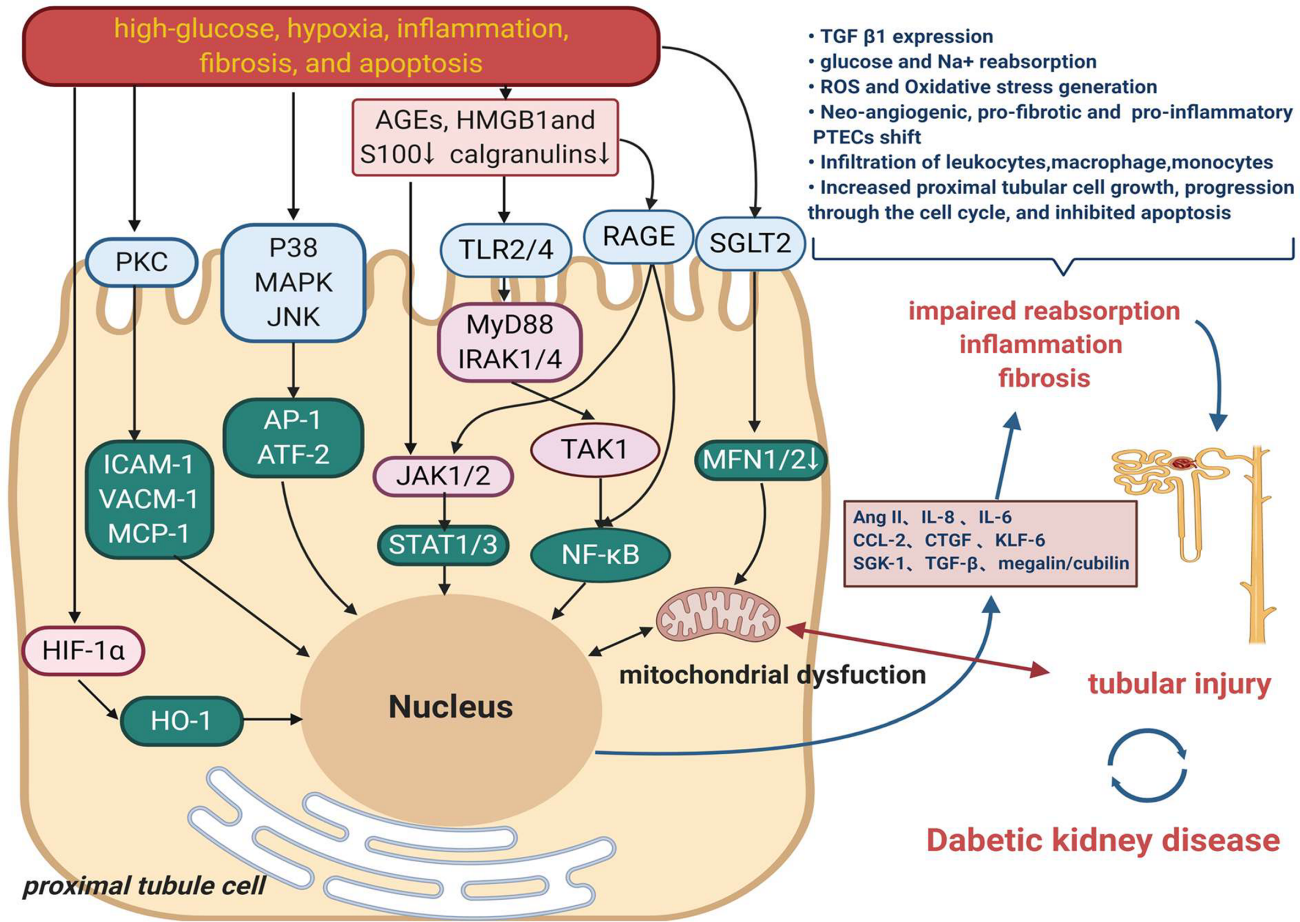
The main mechanism of tubular damage in DKD. Diabetogenic stimuli including high-glucose, oxygen metabolic disorder, inflammation, fibrosis, and apoptosis result in a wide range of injured pathway such as MAPK, PKC signaling. High-mobility group box 1 (HMGB1), s100/calgranulins and advanced glycation end products (AGEs) are danger-associated molecular patterns (DAMPs) that activate cell surface pattern recognition receptors (PRRs), induce signaling events to promote the development of inflammation in DKD. Another mechanism that also might contribute to tubular damage is the increased renal content of HIF1-*α*. Multiple effects on proximal tubule ultimately result in impaired reabsorption, inflammation and fibrosis, which contribute to tubule injury and therefore DKD.

Tubular functional changes in DKD mainly correspond to the modulation of high-glucose, oxygen metabolic disorder, inflammation, fibrosis, and apoptosis (31). **Figure 1** displays the primary mechanism of tubular damage in DKD. Hyperglycemia directly destroys renal tubular cells, resulting in a wide range of cellular and metabolic dysfunctions. Three interrelated and cardinal pathways, including overproduction of reactive oxygen species (ROS), initiation of autophagy, and activation of the apoptotic pathway, are triggered by high glucose and are associated with the progression of DKD (32, 33). Oxidative stress is a state of imbalance in the production of ROS and antioxidant activity in the body, resulting in the activation of downstream inflammation (34) and tubulointerstitial fibrosis-related genes such as transforming growth factor (TGF)-*β*1 and RAAS-related genes (35). Nitric oxide (NO) synthase, xanthine oxidase, nicotinamide adenine dinucleotide phosphate hydrogen (NADPH) oxidase enzymes, and the mitochondrial respiratory chain contribute to kidney ROS generation in a physiological context (36). The pro-oxidant nitrogen oxide (Nox) family members, especially Nox4 and Nox5 isoforms, have been reported to have an important role in the generation of renal ROS in diabetes. Thallas-Bonke V et al. indicated that targeted deletion of NADPH oxidase Nox4 from proximal tubules was dispensable for DKD development (36, 37).

Recent studies stress that the oxygen metabolic disorder which leads to oxidative stress, advanced glycation, hypoxia, and other harmful effects, plays a vital role in renal tubules injury (38, 39). Production and utilization of ATP by the proximal tubular cells are balanced by kidney blood flow, oxygen, and metabolite reabsorption, delivery, and consumption. This balance is now believed to the principal mechanism for regulating tubuloglomerular feedback and maintaining kidney function in diabetes (5, 33). A lately report found that hypoxia-inducible factor-1*α* (HIF-1*α*) activation in tubular cells played an important protective role against diabetic kidney injury by modulation of mitochondrial dynamics through heme oxygenase-1 (HO-1) upregulation, highlighting the potential mechanism and target in DKD (40).

Tubular inflammation is a hallmark of the progression of kidney disease in patients with DM (4). DKD inflammation produces several chemokines, which promote a pro-inflammatory microenvironment and amplify renal injury (41, 42). The majority of the pro-inflammatory responses observed in diabetic kidneys involve the activation of the transcription factor nuclear factor kappa-light-chain-enhancer of activated B cells (NF-*κ*B). The activation of NF-*κ*B and the transcription of certain pro-inflammatory chemokines in tubular epithelial cells are the markers of progressive DKD (43). Gene expression profiling of the tubulointerstitial compartment of patient biopsies has also identified 54 upregulated NF-*κ*B target genes in progressive DKD (44). These studies showed that NF-*κ*B activation stimulated macrophage recruitment and production of inflammatory cytokines [monocyte chemotactic protein-1 (MCP-1)], tumor necrosis factor (TNF)-α, interleukin (IL)-1β, and IL-6) in diabetic kidneys, which were associated with the progression of the disease (45, 46).

In diabetic kidneys, excessive amount of plasma proteins, including albumin, filtered through the damaged glomerulus appears in the glomerular filtrate. Conventional perspectives have emphasized the role of glomerular hypertension and hyperfiltration in the early stage of DKD, which induce the increase in serum creatinine and urinary albumin excretion (47). However, more recent studies have focused on an unchanged glomerular albumin filtration and reduced tubular albumin reabsorption (7, 48). A membrane-associated endocytic receptor megalin (low-density lipoprotein receptor-related protein 2; LRP2) drives the reabsorption of nearly all filtered plasma proteins in cooperation with the receptor protein cubilin (49–51). Protein-overloaded condition occurs in the proximal tubular epithelial cells of the diabetic kidney. Several experimental studies have indicated that protein overload induces proximal tubular cell apoptosis (52), oxidative stress (53), inflammation, and tubulointerstitial fibrosis (54–56). The clinical relevance of increased proximal reabsorption and hyperfiltration in diabetes has been demonstrated by the ability of SGLT2 inhibitors (SGLT2is) to improve renal outcomes in patients with diabetes in large-scale clinical trials, promoting the emergence of the renal tubulocentric hypothesis (15).

### A Link of Diabetogenic Stimuli to Morphological and Functional Changes in Tubules

High levels of glucose-induced oxidative stress contribute to cell death in tubule injury and tubulointerstitial fibrosis in DKD (57). In addition, persistently high levels of glucose can cause abnormal activation of mitochondrial and endoplasmic reticulum stress and intracellular signal transduction pathways, leading to further activation of downstream inflammatory factors and induction of innate immune response (58). The innate immunity in native kidney cells is upregulated at the stage of diabetic microalbuminuria, while tubulointerstitial kidney cell infiltration is associated with albuminuria and fibrosis at a more advanced stage (59). Moreover, it was shown that macrophage accumulation in the interstitium, but not glomeruli, was associated with albuminuria and renal function loss (58). Clustered renal neutrophils were mostly observed in the peritubular space and were associated with accelerated progression and eventual kidney function loss (60). Mast cell accumulation and degranulation were observed in patients with T2DM at varying stages in the periglomerular, peritubular, and perivascular regions of the interstitium. Their presence correlated with tubulointerstitial injury and disease progression (61). These studies suggested that renal tubulointerstitial infiltration by inflammatory cells could accelerate tissue damage. Besides, the components of the glomerular filtrate, such as albumin, advanced glycation end products, growth hormones, *etc.*, interacted with the tubular system and contributed to increased energy consumption, renal oxidative stress, cortical interstitial inflammation, impairment of autophagy, stimulation of hypoxia, and tubulointerstitial fibrosis in DKD (6, 62–64). More convincingly, Vallon et al. illustrated that several diabetogenic stimuli (oxidative stress, tubular renin–angiotensin system, enhanced filtration, and tubular expression of growth factors) induced the growth of the proximal tubules and enhanced tubule reabsorptive capacity, resulting in inflammation, fibrosis, scarring, and impairment of renal function in the diabetic kidney (15).

## Challenges and Progress in the Application of Novel Tubular Biomarkers

In clinical practice, therapeutic strategies for early identification of the kidney lesions in diabetic conditions and consequent slowing of the progression of DKD are still limited and currently mostly rely upon conventional biomarkers. The urine albumin-to-creatinine ratio (uACR) and eGFR are well-standardized and widely used biomarkers for evaluating kidney function and determining different stages of kidney disease in clinical practice. Although carrying prognostic information, eGFR is subject to variation owing to the analytical error of the creatinine measurement and biological variation derived from serum creatinine, patient’s age, and gender (65, 66). ACR, a tubuloglomerular-centric marker, has been recognized as the hallmark of DKD and precedes renal function loss in years. It not only reflects the capacity of glomerular permeability but is also a valuable indicator of tubular damage or dysfunction. The increase in albuminuria followed by glomerular hyperfiltration places a burden on the proximal tubule and elicits an inflammatory response leading to tubulointerstitial damage (67). Nevertheless, a substantial proportion of patients with T1DM or T2DM have renal function impairment without proteinuria, which is known as non-proteinuric DKD (68–70). The data on clinicopathological characteristics, renal prognosis, and all-cause mortality are limited to a handful of clinical trials and longitudinal studies focused on this phenotype. In 2018, the Chronic Renal Insufficiency Cohort (CRIC) study showed that the absence of albuminuria or proteinuria was common and carried a much lower risk for ESKD, chronic kidney disease (CKD) progression, or rapid decline in eGFR than those with albuminuria or proteinuria did (71). In line with this, another propensity score-matched analysis of a nationwide, biopsy-based cohort reported that non-proteinuric DKD patients presented better-controlled blood pressure and fewer typical morphological changes. They were also at a lower risk of CKD progression and all-cause mortality (72). The possible mechanism of developing non-proteinuric DKD may rely on racial/ethnic differences, aging, and response to RAAS inhibitors or other glomerulus-protective drugs before the diagnosis of DKD (68, 73). Therefore, there is still a compelling need to discover potential novel biomarkers for early diagnosis and timely risk stratification in DKD.

**Figure 2:**
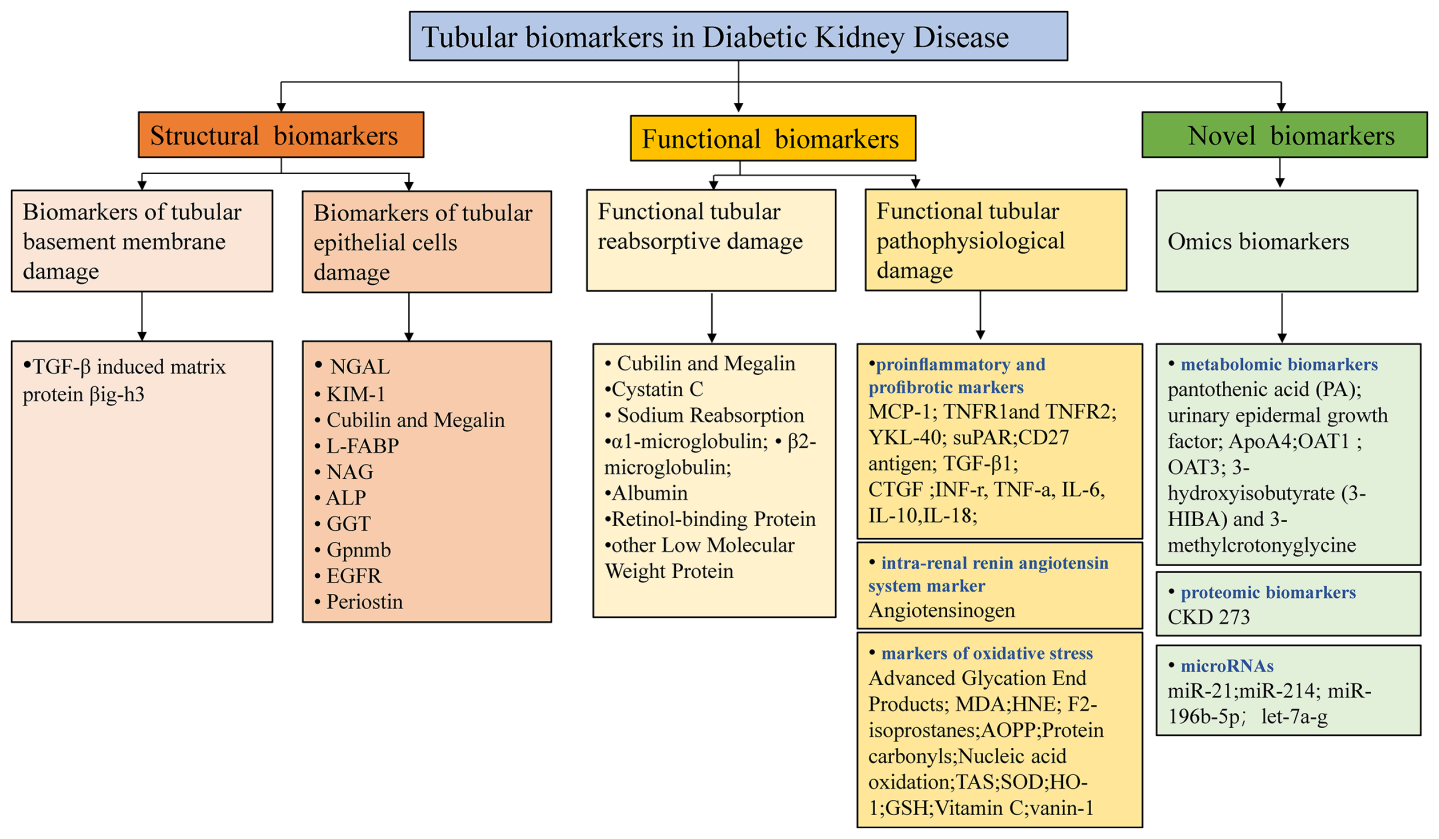
Potential tubular biomarkers in DKD. TGF-*β*, transforming growth factor-*β*; NGAL, neutrophil gelatinase-associated apolipoprotein; KIM-1, kidney injury molecule 1; YKL-40, chitinase-3-like protein 1; MCP-1, monocyte chemoattractant protein-1; L-FABP, liver-type fatty acid binding protein; NAG, N-acetyl-*β*-D-glucosidase; ALP, alkaline phosphatase; GGT, gamma-glutamyl transpeptidase; Gpnmb, glycoprotein Nmb; EGFR, epidermal growth factor receptor; TNFR1/2, tumor necrosis factor receptor 1/2; suPAR, soluble urokinase receptor; CTGF, connective tissue growth factor; INF-*γ*, interferon-*γ*; TNF-α, tumor necrosis factor-α; IL-6/10/18, interleukin 6/10/18; MDA, malondialdehyde; AOPP, advanced oxidation protein products; SOD, superoxide dismutase; HO-1, hemeoxygenase-1; GSH, glutathione; PA, pantothenic acid; OAT1/3, organic anion transporter1/3; 3-HIBA, 3-hydroxyisobutyrate; CKD, chronic kidney disease; TAS, total antioxidant status.

Recent advancements in omics-based biomarkers including proteomics, metabolomics, genome, transcriptome, or lipidome and the integration of these different approaches continue to unveil new potential biomarkers (74). Urinary novel proteomics, peptidomics markers may be associated with impaired proximal tubular reabsorption that almost all of these filtered proteins are reabsorbed into the proximal tubules through megalin/cubilin-mediated endocytosis (75). One study also demonstrated that empagliflozin, the SGLT2i, significantly impacts urinary peptides (76). However, their detection is relatively expensive and still needs time to promote clinical use. Rigorous technical and clinical validation studies are demanded to clarify the specific role and the underlying mechanism. Future research in DKD should attempt to explain how the novel biomarkers can be combined with traditional clinical and biochemical biomarkers in clinical practice to guide screening programs, improve risk stratification, predict response to treatment, and provide a method of monitoring response to treatment. The tubular biomarkers in DKD are summarized in **Figure 2**, which outlines three main classes of the principal tubular biomarkers that may be helpful in early detection and risk-stratification of DKD. The potential applications of these biomarkers in DKD were shown in **Table 1**.

**Table 1:** Summary of principal tubular biomarkers of DKD in clinical use.

Tubular biomarkers	Clinical Importance	Sample	Ref.
**NGAL**	increased when acute tubular damage of various causes occurred; correlated with CKD progression	urine	(77, 78)
	associated with urinary albumin excretion, rapid decline of eGFR, and increased serum creatinine	urine	(79–82)
	associated with renal progression to ESKD, progressive tubular structural and functional impairment	urine	(10, 83–85)
	best predictive cutoff value of urinary NGAL to creatine ratio (uNCR) for T2DKD diagnosis was 60.685 ng/mg;	urine	(82)
	7.595 times higher risk of nephrotic-range proteinuria in T2DKD patients with uNCR >60.685 *vs.*≤60.685 ng/mg.		
	twofold or greater risk for CKD progression in patients with diabetes;	urine	(10)
	1.5-fold or greater risk for CKD progression in patients without diabetes		
**KIM-1**	repaired injury by removing apoptotic bodies and cellular debris	urine	(8)
	upregulated when kidney damages	urine	(86)
	largely restricted to tubular cells in areas with tubulointerstitial damage induced by overload proteinuria; upregulated in proteinuric nephropathy and associated with renal fibrosis and inflammation.	tissue	(55) (87)
	elevated in T2DM with normal or mildly increased albuminuria	urine	(88)
	increased in T1DM patients who developed from macroalbuminuria to late-stage CKD	urine	(89)
	elevated in the high-risk group which was stratified by both ACR and eGFR; decreased in the very high-risk group; not associated with either eGFR or albuminuria	urine	(84)
	no predictive value for progression to ESKD independently of albumin excretion rate (AER); no prognostic benefit to conventional biomarkers (AER, eGFR); causal impact of KIM-1 on the decrease of eGFR in T1DM by Mendelian randomization analysis	urine	(89)
	no association with uKIM-1-to-creatinine ratio and eGFR decline in patients with T2DM	urine	(13)
	contains most of the predictive information for eGFR progression in T1DM	urine	(90)
	predictive value for the rapid decline of renal function in DKD	urine/serum	(81, 91, 92)
	associated with DKD progression and yearly decline in eGFR	plasma	(9)
	the most important predictor by cross-omics technologies	urine	(93)
**YKL-40**	a marker of inflammation and endothelial dysfunction; an indicator of tubular injury severity	/	(94, 95)
	associated with albuminuria in T1DM and in early stage of nephropathy in T2DM	plasma	(96) (13, 94, 97)
	elevated among macroalbuminuric T2DM patients	urine	(98)
	not associated with eGFR decline and varying levels of baseline eGFR and albuminuria in T2DM	plasma	(99)
	a plasma marker of DKD progression	plasma	(9)
**MCP-1**	upregulated and expressed in the diabetic glomerular and renal tubular epithelium	urine	(100)
	correlated with the extent of interstitial inflammatory infiltrate	urine	(101, 102)
	associated with severity of proteinuria in DKD	urine	(103)
	elevation in renal tubuli contributes to renal tubular damage in DKD	tissue	(103)
	MCP-1-to-creatinine ratio concentrations were strongly associated with sustained renal decline, severity of kidney damage in T2DM	urine	(13) (84)
	associated with an increased risk of DKD progression only among patients with baseline eGFR<45 ml/min per 1.73 m^2^	plasma	(9)
**Cubilin and megalin**	increased in microalbuminuria groups compared with non-albuminuric groups in T1DM	urine	(104)
	genetic association exists between a cubilin and a rare megalin variant with diabetes-associated ESKD in populations with recent African ancestry	gene	(105)
	upregulated renal megalin expression in early T2DM rats	tissue	(106)
	elevated in two models of insulin-deficient diabetes in drug-inducible megalin knockout mice	tissue	(107)
	megalin in both segment 1 and segment 2 participated in clearing the ultrafiltrate from proteins in both cortical and juxtamedullary nephrons under normal conditions	tissue	(108)
	megalin in segment 3 was inactive with regard to protein endocytosis; it was activated by the presence of proteins in the lumen of the tubule in normal physiology	tissue	(108)

NGAL, neutrophil gelatinase-associated apolipoprotein; KIM-1, kidney injury molecule 1; YKL-40, chitinase-3-like protein 1; MCP-1, monocyte chemoattractant protein-1; T1DM/T2DM, type 1/2 diabetes mellitus; CKD, chronic kidney disease; ESKD, end-stage kidney disease; DKD, diabetic kidney disease; eGFR, estimated glomerular filtration rate; AER, albumin excretion rate; ESKD, end-stage of kidney disease; uNCR urinary NGAL to creatine ratio.

### Neutrophil Gelatinase-Associated Lipocalin

NGAL is a 24 kDa secreted glycoprotein that belongs to the lipocalin protein family. As mainly released by neutrophils and distal tubular cells, it rapidly increases when acute tubular damage of various causes occurs (109). Following the discovery that NGAL levels are also raised in the CKD setting, this marker has been suggested to correlate with CKD progression (77, 78). More importantly, a great number of studies have demonstrated the important role of NGAL in predicting the evolution of DKD. In a study of T2DM patients and healthy controls, Fu et al. reported that NGAL increased across the four groups from controls to normoalbuminuric, microalbuminuric, and macroalbuminuric patients (79). In several observational single-center follow-up studies, elevated urine NGAL level was shown to be associated with urinary albumin excretion (80), the rapid decline in eGFR and increased serum creatinine (81), renal progression to ESKD (83), and progressive tubular structural and functional impairment (84). Consistently, our cohort study found that the best predictive cutoff value of urinary NGAL to creatine ratio (uNCR) for DKD diagnosis was 60.685 ng/mg, and T2DM patients with the increased level of uNCR had a higher risk of nephrotic-range proteinuria and worse renal outcome (82). Furthermore, a more recent report from the CRIC study conducted at seven US clinical centers provided solid evidence that higher urinary NGAL levels were not only strongly associated with cardiac markers, but were also linked to an approximately twofold or greater risk of CKD progression in patients with DM (10). It has been postulated that NGAL captures some of the variability in the rate of kidney function decline independently of albuminuria or other risk factors and reflects tubular injury and inflammation in the setting of DKD (10, 85).

### Kidney Injury Molecule 1

KIM-1 is a transmembrane protein expressed on the apical membrane of proximal tubule cells (110). KIM-1 facilitates the repair of the injury by removing apoptotic bodies and cellular debris from the damaged tubulointerstitial compartment (8). Han et al. reported that urinary KIM-1 was not detectable in normal kidneys while its levels were upregulated with the occurrence of kidney injury (86). Consistently, renal KIM-1 expression was largely restricted to tubular cells in areas with tubulointerstitial damage in an experimental model of tubulointerstitial damage induced by overload proteinuria (55), and it was also upregulated in patients with proteinuric nephropathy (87). Hence, KIM-1 was suggested to be a specific and sensitive biomarker of proximal tubular damage. However, there has been a controversy about the changes in its serum and urine levels, as well as its association with kidney progression in DKD. In several studies, urine KIM-1 was elevated in T2DM patients with normal or mildly increased albuminuria (88) and in T1DM patients who developed from macroalbuminuria to late-stage CKD (89). However, Siddiqui et al. found that urinary KIM-1 was elevated in the high-risk group (stratified by both ACR and eGFR) and reduced in the very high-risk group. Also, it was not found to be associated with either eGFR or albuminuria (84). The disparity of those studies may be due to the limited sample sizes and selected population. In a large-sample randomized-controlled trial in T1DM conducted by Panduru et al., KIM-1 had no predictive value for progression to ESKD independently of albumin excretion rate (AER) and added no prognostic benefit to conventional biomarkers (AER, eGFR). However, the causal impact of KIM-1 on the decrease of eGFR in T1DM was confirmed by Mendelian randomization analysis (89). Nadkarni et al. did not find any association with uKIM-1-to-creatinine ratio and eGFR decline in patients with T2DM and preserved renal function from the ACCORD Trial population (13). Another recent report in T1DM patients from the Scottish Diabetes Research Network Type 1 Bioresource (SDRNT1BIO) and the Finnish Diabetic Nephropathy (FinnDiane) study showed that just the serum KIM-1, as well as CD27, contained most of the predictive information for eGFR progression among a large set of associated biomarkers evaluated with the Luminex platform and LC electrospray tandem MS (LC-MS/MS) (90). More recent evidence still emphasizes the important role of KIM-1 in DKD. In 2020, a multicenter and prospective cohort within the CRIC Study suggested that higher plasma KIM-1 levels were associated with DKD progression and yearly decline in eGFR (9). Kammer et al. reported that the discrimination of eGFR trajectories in individuals with the incident or early DKD and maintained baseline eGFR was modest, and KIM-1 was the most critical predictor by cross-omics technologies (93).

### YKL-40

YKL-40, which is composed of three N terminal amino acids tyrosine (Y), lysine (K), and leucine (L), is a low-molecular-weight (40 kDa) heparin- and chitin-binding glycoprotein. Also known as cartilage glycoprotein-39 or chitinase 3-like protein 1 (CHI3L1), YKL-40 is a product of the chitinase 3-like 1 gene and a growth factor for several cell types. It has an established role in extracellular matrix remodeling and angiogenesis (111). Moreover, YKL-40 acts as a marker of inflammation and endothelial dysfunction. It is secreted by various cells such as neutrophils and activated macrophages in different inflamed tissues and vascular smooth muscle cells (94). Increasing evidence stressed the role of YKL-40 in kidney disease. YKL-40 was demonstrated to be an indicator of tubular injury severity, and it was upregulated in kidney macrophages after ischemia–reperfusion injury (95). It played a role in limiting tubular cell apoptosis during the repair phase of acute kidney injury (AKI) (95). The association of YKL-40 with DKD has also been suggested. Several studies have suggested that urine YKL-40 has a limited role. In contrast, plasma YKL-40 was independently associated with albuminuria in T1DM and in the early stage of nephropathy in T2DM patients (13, 94, 96, 97). However, one study documented that urinary excretion of YKL-40 was significantly elevated among macroalbuminuric T2DM patients (98), while another study reported that plasma YKL-40 was not associated with eGFR decline in participants with type 2 diabetes and varying levels of baseline eGFR (mean eGFR 78 ml/min per 1.73 m^2^) and albuminuria (99). More convincing results were obtained from a multicenter, prospective, large-sample cohort within the CRIC Study, providing new insights on YKL-40 as a plasma marker of DKD progression. Increased plasma YKL-40 concentrations were associated with DKD progression and decline in eGFR over time, even after adjustment for potential confounders and other plasma biomarkers (9).

### Monocyte Chemoattractant Protein-1

MCP-1 (or C-C chemokine ligand 2) is a member of the C-C chemokine family, recruiting monocytes and influencing macrophage accumulation (112, 113). As an inflammatory biomarker, MCP-1 is highly upregulated in the diabetic glomerular and tubular epithelium (100). Previous studies have documented that urinary MCP-1 levels not only correlate with the extent of interstitial inflammatory infiltration but also are associated with the development of albuminuria and renal damage (101, 114). Morii et al. found that MCP-1 was produced in renal tubular cells and released into the urine in proportion to the degree of albuminuria. Increased renal tubular MCP-1 expression contributed to tubular damage in DKD (103). The ACCORD trial enrolled 10,251 T2DM patients with preserved renal function and examined the association of four biomarker-to-creatinine ratio levels; only MCP-1-to-creatinine ratio concentrations were strongly associated with the sustained renal decline (13). Siddiqui et al. also found that elevated urinary MCP-1 was related to the severity of kidney damage, and it was expressed more in progressive renal impairment in T2DM (84). The 2020 CRIC Study first reported an association of plasma MCP-1 concentrations and DKD progression among individuals with moderate to severe kidney disease. Higher plasma MCP-1 levels were associated with an increased risk of DKD progression only among patients with baseline eGFR<45 ml/min per 1.73 m^2^ (9).

### Cubilin and Megalin

In physiological conditions, proximal tubule epithelial cells have the capacity of reabsorbing nearly all low-molecular-weight serum proteins and ultrafiltrated albumin, along with glucose, phosphate, amino acids, and various ions. The key contributor for the uptake ability of the epithelial cells essentially relies on the collective effort of two apical membrane receptors cubilin (CUBN) and megalin (LRP2), which form a complex expressed at the brush border (115). Both cubilin and megalin are huge multiligand receptors (460 and 600 kDa, respectively), each of which could independently bind to an amount of identified substrates including albumin and vitamin D binding protein (VDBP) (49). After ligand binding, cubilin/megalin ligands interact and are internalized to proximal tubular epithelial cells’ (PTECs) endosomes and lysosomes for catabolic degradation and receptor recycling (116). Using a GeLC/MS platform proteomics approach, Thrailkill et al. first propose that enhanced cubilin and megalin excretion might serve as important markers of DKD, considering that urinary cubilin and megalin were significantly higher in microalbuminuria groups than in non-albuminuric groups in T1DM patients (104). Both album infiltration and reabsorption were observed elevated in two models of insulin-deficient diabetes and drug-inducible megalin knockout mice (107). A study published in 2020 explained that megalin in both segment 1 and segment 2 participated in clearing the ultrafiltrate from proteins in both cortical and juxtamedullary nephrons under normal conditions. Although megalin in segment 3 was inactive concerning protein endocytosis, it was activated by the presence of proteins in the lumen of the tubule in normal physiological conditions (108). These studies provided a theoretical rationale and backbone for early treatment to improve the capacity of proximal tubule to avoid the development of proteinuria.

## Renal Tubule-Targeting Therapeutics: A New Era for DKD Management

**Figure 3:**
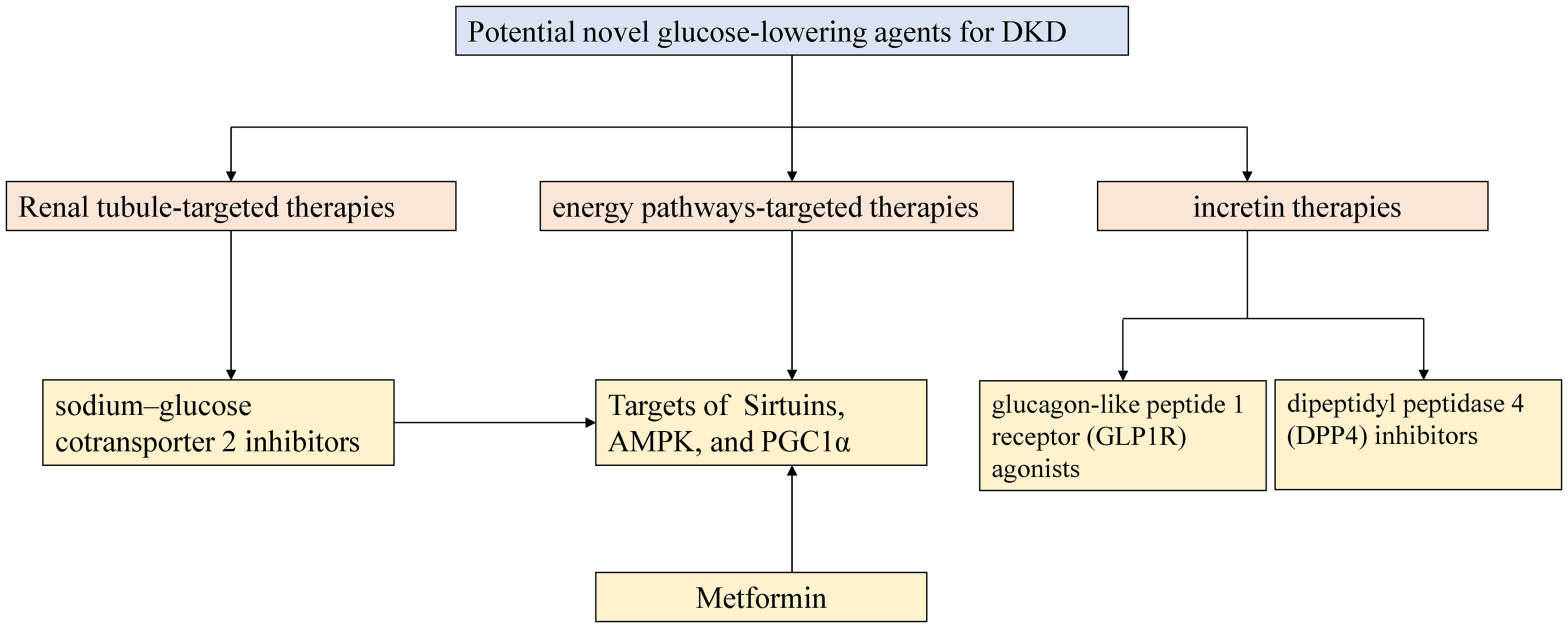
Outlines of potential novel glucose-lowering agents for DKD. AMPK 5-AMP-activated protein kinase; PGC-1*α* peroxisome proliferator-activated receptor *γ* coactivator-1 alpha.

In addition to the new tubulocentric insights for DKD mentioned above, the emergence of new anti-hyperglycemic agents has considerably altered the therapeutic landscape of DKD. For decades, the cornerstone of DKD therapeutics relied on lifestyle interventions, strategies for hyperglycemia and hypertension in combination with the use of angiotensin-converting enzyme inhibitors (ACEIs) or angiotensin receptor blockers (ARBs) (117). Recent advances in studies on novel glucose-lowering agents promote the new era in the advanced glycemic control and concurrently promise cardiorenal protection in DKD management. **Figure 3** depicts the current high-profile classes of potential novel anti-hyperglycemic agents for DKD, mainly grouped into renal tubule-targeting therapies, incretin therapies, and energy pathways-targeting therapies (117, 118). The tubule-targeting medicine, SGLT2i also affects the energy pathway associated with enhanced sirtuin1 and hypoxia-inducible factor (HIF)-2a signaling (119). In addition to SGLT2i, incretin drugs include glucagon-like peptide 1 receptor (GLP1R) agonists and dipeptidyl peptidase 4 (DPP4) inhibitors, which also have the potential to improve tubulointerstitial function. GLP1R expression was detected in macrophages, endothelial cells, juxtaglomerular cells, and proximal tubules within the kidney in various animal models and human tissue (117). Endogenous GLP1R signaling exerts a natriuretic action in DKD. Direct GLP1R-stimulation induces diuresis and natriuresis by increasing GFR and inhibiting the activity of the sodium-hydrogen exchanger isoform 3 (NHE3) in the proximal tubule (120, 121). Nevertheless, DPP4 inhibitors demonstrate modest kidney-protective effects. Compared with the GLP1R agonists, they mainly attenuate albuminuria without an impact on eGFR decline. DPP4 inhibitors indirectly modulate glucose-dependent insulin secretion and suppress glucagon secretion from pancreatic *α*-cells by elevating endogenous GLP1 levels (122). Linagliptin, the only available DPP4 inhibitor, showed a significant improvement in albuminuria progression but not in kidney outcomes in the Cardiovascular and Renal Microvascular Outcome Study with Linagliptin (CARMELINA) trial (123). No significant placebo-adjusted changes in eGFR or albuminuria with linagliptin therapy were observed in the Modification of Albuminuria in T2D and CKD with the LINAgliptin (MARLINA-T2D™) study (124).

Among diabetic medications, SGLT2i attracts considerable attention for their pleiotropic effects on glycemic control, renal protection, cardiovascular benefits, blood pressure control, and attenuation of lipid levels. SGLT2 is a low-capacity and high-affinity glucose transporter with 1:1 Na+/glucose stoichiometry. It is located in the S1–2 segment of the proximal convoluted tubules and is responsible for reabsorption of 90% of glucose filtered through the glomerulus (125). Multiple mechanisms are explored involving the kidney protection of SGLT2 inhibition, mainly characterized into (1) attenuation of proximal tubular oxidative stress, mitochondrial morphology, modulation of key metabolism and reabsorptive proteins, pro-inflammatory and profibrotic cytokines, and improvement of tubulointerstitial fibrosis; (2) through activation of tubuloglomerular feedback to regulate glomerular hemodynamic stability and metabolic effects (126, 127). **Table 2** summarizes the underlying mechanism of kidney protection by SGLT2 inhibition in DM reported in recent years.

**Table 2:** Proposed hypotheses for the kidney protective mechanisms of SGLT2 inhibitors in DKD.

Mechanisms	Ref.
decreased sodium uptake by Na^+^/H^+^ exchanger isoform 3 (NHE3) expression in proximal convoluted tubules (PTs)	(128–133)
reduced urinary excretion of angiotensin II and angiotensinogen levels in SGLT2 inhibitor-treated T2DM rats	(134)
did not further activate RAS in the long term, which prevented the RAS-mediated aggravation of cardiovascular and renal events	(134, 135)
reduced urinary angiotensinogen excretion in patients with T2DM	(136)
increased urinary angiotensinogen excretion in patients with T1DM	(137, 138)
modulated the tubular expression of proteins governing the medullary concentration activity, further had an effect on fluid and electrolyte balance	(139, 140) (132)
blocked the activation of the apoptotic-associated protein within PT cells	(141)
glomerular fibrosis or injury was not alleviated in SGLT2-knockout diabetic mice	(142)
modulated oxidative stress and intraglomerular inflammation and could thus alleviate renal fibrosis	(143)
alleviated the generation of vanin-1, the biomarker for oxidative stress within the kidney	(144)
lessened the epithelial-to-mesenchymal transition by modulating miR21	(145)
alleviated renal fibrosis by lowering lipid accumulation-induced inflammation mediated by CD68 macrophages	(146)
activation of tubuloglomerular feedback: alleviated apoptosis by increasing autophagosomal formation within glomerular mesangial cells and podocytes	(147, 148)
anti-inflammatory effects: decreased the levels of several cytokines such as tumor necrosis factorα (TNFα), interleukin-6, high-sensitivity C-reactive protein, and leptin	(149, 150)
restored oxygen supply, thereby alleviating the metabolic stress state in the mitochondria and restoring the hematocrit level in patients with DM	(151, 152)
reduced ECM fibrosis by inflammation reduction and RAAS overactivation	(153)
the EPO-producing ability in patients with DM might be reversed after treatment with SGLT2i	(154)
suppressed HIF-1*α*-mediated metabolic switch from lipid oxidation to glycolysis in kidney tubule cells of diabetic mice.	(155)
inhibited aberrant glycolytic metabolism and mitochondrial ROS formation in PTEC in high-glucose conditions.	(156)
*via* the reduction of megalin O-GlcNAcylation and the following megalin internalization and endocytic functional suppression to attenuate protein overload in renal proximal tubule in progressive DKD.	(56)
promoted elevation of ketone bodies, which subsequently inhibited mTORC1 in the proximal renal tubules, explaining their protective effects s in non-proteinuric and proteinuric DKD.	(157)
Empagliflozin protected against proximal renal tubular cell injury induced by high glucose *via* regulation of hypoxia-inducible factor 1-alpha.	(158)

NHE3, Na+/H+ exchanger isoform 3; PT, proximal convoluted tubule; SGLT2, sodium-glucose co-transporter 2; T1DM/T2DM, type 1/2 diabetes mellitus; RAS, renin-angiotensin system; RAAS, Renin-angiotensin-aldosterone System; TNFα, tumor necrosis factorα; ECM, extracellular matrix; EPO, erythropoietin; DM, diabetes mellitus; HIF-1α, hypoxia inducible factor-1α; PTEC, Proximal Tubular Epithelial Cell; DKD, Diabetic Kidney Disease; mTORC1, mammalian target of rapamycin complex 1.

There is increasing evidence suggesting that SGLT2i has renal protective effects in addition to cardiovascular protection, as reported by diverse clinical trials (summarized in **Table 3**). The first clues involving the potential nephroprotection with SGLT-2 inhibitors originated from glucose-lowering trials that set albuminuria as a secondary outcome (167). In the Empagliflozin Cardiovascular Outcome Event (EMPA-REG OUTCOME) trial, the treatment of empagliflozin significantly reduced the primary end points which were defined as progression to macroalbuminuria, doubling of the serum creatinine level (D-Scr), initiation of kidney replacement therapy, or renal death, and incident albuminuria (159) (**Table 3**). In addition, all individual renal end points showed notable attenuation (31, 168). In the subsequent published Canagliflozin Cardiovascular Assessment Study (CANVAS) and CANVAS-Renal (R) program studies, clear renal protective effects were also noted (160, 161). Kidney function declined in a relatively stable manner, and urine albumin loss decreased in participants who received canagliflozin *vs.* placebo. Regarding the Dapagliflozin Effect on Cardiovascular Events-Thrombolysis in Myocardial Infarction 58 (DECLARE-TIMI 58) trials, although treatment with dapagliflozin showed a non-inferior rate of major adverse cardiovascular events (MACEs) than placebo, a possible lower rate of adverse renal outcomes in the dapagliflozin group than in the placebo group was observed (163). Although the above cardiovascular trials indicated nephroprotective effects of SGLT2i, it should be noted that the recruitment of participants was biased, considering that the selected patients had a high risk of cardiovascular events and mostly normal kidney function (169). Canagliflozin and Renal Events in Diabetes with Established Nephropathy Clinical Evaluation(CREDENCE) was the first dedicated renal outcomes trial of an SGLT2i canagliflozin, the recruitment of which was randomized in 4,401 T2DM patients with CKD, severely elevated albuminuria, and already ACEIs or ARBs receivers (162). The incidence rates of primary composite outcomes (D-Scr, ESKD or renal/CV death) and the renal-specific composite outcomes (D-Scr, ESKD or renal death) were significantly lower in the canagliflozin group than in the placebo group. Subsequently, two trials embarked on investigating the kidney effects of SGLT-2 inhibitors in CKD patients with or without DM (169). The Dapagliflozin and Prevention of Adverse Outcomes in Chronic Kidney Disease (DAPA-CKD) trial enrolled 4,304 CKD patients with an eGFR ranging from 25 to 75 ml/min/1.73 m², and uACR range from 200 to 5,000 mg/g (164). The trial aimed to evaluate the effect of dapagliflozin 10 mg once daily compared with placebo in addition to a maximum tolerated labeled dose of an ACEI or ARB. Reductions of the same magnitude in the primary outcomes (a composite of a sustained decline in the estimated GFR of at least 50%, ESKD, or renal/CV death) and renal-specific composite outcomes (D-Scr, ESKD, or renal death) were noted. The benefit was comparable for patients with diabetic and non-diabetic CKD. The Heart and Kidney Protection with Empagliflozin (EMPA-KIDNEY) trial commenced in November 2018, with a plan to recruit 5,000 participants and to be completed in June 2022 (170). The empagliflozin on estimated extracellular volume, estimated plasma volume, and measured glomerular filtration rate in patients with heart failure (Empire HF Renal) trial focused on the effects of empagliflozin in both heart failure and CKD patients. It enrolled 391 patients with left ventricular ejection fraction (LVEF) ≤40% and eGFR >30 ml/min/1.73 m². The results showed that empagliflozin reduced estimated extracellular volume, estimated plasma volume, and measured GFR after 12 weeks, implying that fluid volume changes might be an important mechanism underlying the beneficial clinical effects of SGLT2i (165). However, the recent Evaluation of Ertugliflozin Efficacy and Safety Cardiovascular Outcomes Trial (VERTIS CV) reported no significant benefit of ertugliflozin for the renal composite outcomes (death from renal causes, renal replacement therapy, or D-Scr) (166). Further analyses in the trial using renal different end points are underway and may give more clues. To sum up, both in the cardiovascular outcomes trials, which set different definitions of renal outcomes as secondary end points, and in the dedicated trials in CKD patients in which cardiorenal composite outcomes were primary end points, SGLT2i mostly displayed a convincing significant hindering of kidney progression.

**Table 3:** Summary of the main renal outcomes of the SGLT2 inhibitors trials.

Trial name/drug	Study population	Primary endpoint	Renal outcomes	Effect size (SGLT2i *vs*. placebo)	Renal benefits *vs*. placebo	Ref.
**the EMPA-REG OUTCOME/empagliflozin**	7,020 T2DM, established cardiovascular disease, with eGFR >30 ml/min/1.73 m²	progression to macroalbuminuria D-Scr, initiation of KRT, or death from renal disease, and incident albuminuria	Doubling of Scr with eGFR ≤45 ml/min/1.73 m2, initiation of KRT, or renal death	HR 0.54 (95%CI 0.40–0.75)	Superior	(159)
			Incident or worsening nephropathy	HR 0.61(95%CI 0.53–0.70)		
**the CANVAS Program/Canagliflozin**	10,142 T2DM, high cardiovascular risk, with eGFR >30 (ml/min/1.73 m²)	a composite of death from cardiovascular causes, non-fatal myocardial infarction, or nonfatal stroke	At least 40% reduction in eGFR, need for KRT, or renal death	HR 0.60 (95%CI 0.47–0.77)	Superior	(160)
			Progression of albuminuria	HR 0.73 (95% CI, 0.67–0.79		
**the CANVAS-R Program/Canagliflozin**	10,142 T2DM	a composite of sustained and adjudicated D-Scr, ESKD, or renal death	D-Scr, ESKD, or renal death	HR 0.53 (95% CI 0·33–0·84)	Superior	(161)
			40% reduction in eGFR, ESKD, or death from renal causes	HR 0.60 (95% CI 0·47–0·77)		
**the CREDENCE Trial/Canagliflozin**	4,401 T2DM and albuminuric CKD	D-Scr, ESKD, or renal/CV death	D-Scr, ESKD, or renal/CV death	HR 0.70 (95% CI, 0.59–0.82)	Superior	(162)
			D-Scr, ESKD, or renal death	HR 0.66 (95% CI, 0.53–0.81)		
**the DECLARE-TIMI 58/Dapagliflozin**	17,160 T2DM	MACE and a composite of cardiovascular death or hospitalization for heart failure	At least 40% reduction in eGFR to less than 60 ml/min per 1.73 m^2^, ESKD, or renal/CV death	HR 0.76 (95% CI 0.67–0.87)	Superior	(163)
			At least 40% reduction in eGFR to less than 60 ml/min per 1.73 m^2^, ESKD, or renal death	HR 0.53 (95% CI 0.43–0.66)		
**DAPD-CKD**	4304 CKD, with eGFR25-75(ml/min/1.73 m²), uACR 200 to 5,000 mg/g	a composite of a sustained decline in the estimated GFR of at least 50%, ESKD, or renal/CV death	Primary outcome	HR 0.61 (95% CI 0.51–0.72)	Superior	(164)
			Renal-specific composite outcome (D-SCr, ESKD, or renal death)	HR 0.56 (95% CI, 0.45–0.68)		
**Empire HF Renal trial/Empagliflozin**	391 heart failure patients, LVEF <=40%, with eGFR >30(ml/min/1.73 m²)	the between-group difference in the changes in estimated extracellular volume, estimated plasma volume, and measured GFR from baseline to 12 weeks.	Primary outcomes	reductions in estimated extracellular volume (adjusted mean difference −0.12 L, 95% CI −0.18 to −0.05; p = 0.00056), estimated plasma volume (−7.3%, −10.3 to −4.3; p < 0·0001), and measured GFR (−7.5 ml/min, −11.2 to −3.8; p = 0.00010)	Superior in Fluid volume changes	(165)
**VERTIS CV trial/ertugliflozin**	8,246 patients with type 2 diabetes and established atherosclerotic cardiovascular disease	a composite of death from cardiovascular causes, nonfatal myocardial infarction, or nonfatal stroke (*i.e*., a major adverse cardiovascular event).	renal-specific composite outcome (D-SCr, ESKD, or renal death)	HR 0.81 (95.8% CI, 0.63 to 1.04)	No significant benefit	(166)

D-Scr, doubling of the serum creatinine level; KRT, kidney replacement therapy; ESKD, end-stage of kidney disease; LVEF, left ventricular ejection fraction; MACEs, major adverse cardiovascular events defined as cardiovascular death, myocardial infarction, or ischemic stroke; uACR, urinary albumin-to-creatinine ratio (with albumin measured in milligrams and creatinine measured in grams); HR, hazard ratio.

These impressive clinical trials and mechanistic studies of SGLT2i promoted the clinical guidelines and recommendations to update the optimal approaches for the prevention and management of DKD. In 2019, the American Diabetes Association (ADA), European Association for the Study of Diabetes (EASD), and European Society of Cardiology (ESC) published updated recommendations for the management of patients with T2DM and a high cardiovascular risk, highlighting the cardiorenal benefits of SGLT2i and glucagon-like peptide-1 receptor agonists (GLP-1 RA) (171–174). The ESC guidelines suggest that SGLT2i or GLP­1 receptor agonists should have priority when patients coexist with cardiovascular disease and those at high or very high cardiovascular risk. Likewise, the ADA-EASD consensus report indicates that patients at high risk of cardiorenal disease are recommended to be treated with SGLT2i or GLP­1 receptor agonists, independent of glycosylated hemoglobin (HbA1c) levels. Additionally, SGLT2i, as well as metformin, was recommended as first-line glycemic management for patients with T2D and CKD according to the 2020 Kidney Disease Improving Global Outcomes (KDIGO) guideline for diabetes management in CKD, in light of the kidney benefits for most patients with eGFR ≥30 ml/min per 1.73 m^2^ (175). Empagliflozin and canagliflozin are FDA-approved for use in patients with eGFR ≥45 ml/min/1.73 m^2^, and ertugliflozin and dapagliflozin are used for those with eGFR ≥60 ml/min/1.73 m^2^ (166, 176).

## Future Perspectives

Great research progress in understanding the pathogenesis of tubular damage and novel biomarkers and treatments has been made, promoting us the transition into a new era of personalized diagnosis and therapy in DKD. As a complex and major complication of metabolism disease, diabetic tubular dysfunction should be regarded with close interconnection with glomerular changes and compact interrelation with systemic metabolic changes. The major current challenges in discovered biomarkers in DKD include the integration of clinical and biochemical biomarkers and omic biomarkers and translation into the pathophysiology, differential diagnosis, risk stratification, prognosis, and individual therapy in clinical practice. The ongoing progress with new anti-hyperglycemic agents provides invaluable and novel insights into the pathophysiology and potential biomarkers of renal tubules in DKD, the combination of which will shed light on better clinical management of DKD.

## Author Contributions

SD drafted the manuscript, designed the figures and tables. FL and DS corrected the figures and tables. CZ and BZ reviewed the draft. CX was responsible for the final substance. YY was the guarantor and supervised the review and edited the review. All authors contributed to the article and approved the submitted version.

## Funding

This work was supported by grants from the National Natural Science Foundation of China (No. 81870469, 81670628, 81300573), the Natural Science Foundation of Jiangsu Province (No. BK20131030 to Yanggang Yuan, BK20191075 to Suyan Duan), the China Scholarship Council (CSC, File No. 201608320124), Chinese Society of Nephrology (17010060675 to Yanggang Yuan, 17010090678 to Suyan Duan), the Clinic Research Center of Jiangsu Province (No. BL2014080) and the Priority Academic Program Development of Jiangsu Higher Education Institutions.

## Conflict of Interest

The authors declare that the research was conducted in the absence of any commercial or financial relationships that could be construed as a potential conflict of interest.

## References

[1] Afkarian M Zelnick LR Hall YN Heagerty PJ Tuttle K Weiss NS. Clinical Manifestations of Kidney Disease Among Us Adults With Diabetes, 1988-2014. Jama (2016) 316:602–10. 10.1001/jama.2016.10924 PMC5444809 27532915

[2] Fu H Liu S Bastacky SI Wang X Tian XJ Zhou D. Diabetic Kidney Diseases Revisited: A New Perspective for a New Era. Mol Metab (2019) 30:250–63. 10.1016/j.molmet.2019.10.005 PMC6838932 31767176

[3] Zou LX Sun L. Global Diabetic Kidney Disease Research From 2000 to 2017: A Bibliometric Analysis. Medicine (2019) 98:e14394. 10.1097/MD.0000000000014394 30732183 PMC6380778

[4] Tang SC Lai KN. The Pathogenic Role of the Renal Proximal Tubular Cell in Diabetic Nephropathy. Nephrol Dial Transplant (2012) 27:3049–56. 10.1093/ndt/gfs260 22734110

[5] Gilbert RE. Proximal Tubulopathy: Prime Mover and Key Therapeutic Target in Diabetic Kidney Disease. Diabetes (2017) 66:791–800. 10.2337/db16-0796 28325740

[6] Thomas MC Burns WC Cooper ME. Tubular Changes in Early Diabetic Nephropathy. Adv Chronic Kidney Dis (2005) 12:177–86. 10.1053/j.ackd.2005.01.008 15822053

[7] Russo LM Sandoval RM Campos SB Molitoris BA Comper WD Brown D. Impaired Tubular Uptake Explains Albuminuria in Early Diabetic Nephropathy. J Am Soc Nephrol JASN (2009) 20:489–94. 10.1681/ASN.2008050503 PMC2653682 19118149

[8] Zeni L Norden AGW Cancarini G Unwin RJ. A More Tubulocentric View of Diabetic Kidney Disease. J Nephrol (2017) 30:701–17. 10.1007/s40620-017-0423-9 PMC5698396 28840540

[9] Schrauben SJ Shou H Zhang X Anderson AH Bonventre JV Chen J. Association of Multiple Plasma Biomarker Concentrations With Progression of Prevalent Diabetic Kidney Disease: Findings From the Chronic Renal Insufficiency Cohort (Cric) Study. J Am Soc Nephrol JASN (2021) 32:115–26. 10.1681/ASN.2020040487 PMC7894671 33122288

[10] Anderson AH Xie D Wang X Baudier RL Orlandi P Appel LJ. Novel Risk Factors for Progression of Diabetic and Nondiabetic Ckd: Findings From the Chronic Renal Insufficiency Cohort (Cric) Study. Am J Kidney Dis Off J Natl Kidney Foundation (2021) 77:56–73.e1. 10.1053/j.ajkd.2020.07.011 PMC7752839 32866540

[11] Satirapoj B Nast CC Adler SG. Novel Insights Into the Relationship Between Glomerular Pathology and Progressive Kidney Disease. Adv Chronic Kidney Dis (2012) 19:93–100. 10.1053/j.ackd.2011.12.001 22449346

[12] Dunkler D Gao P Lee SF Heinze G Clase CM Tobe S. Risk Prediction for Early CKD in Type 2 Diabetes. Clin J Am Soc Nephrol CJASN (2015) 10:1371–9. 10.2215/CJN.10321014 PMC4527032 26175542

[13] Nadkarni GN Rao V Ismail-Beigi F Fonseca VA Shah SV Simonson MS. Association of Urinary Biomarkers of Inflammation, Injury, and Fibrosis With Renal Function Decline: The ACCORD Trial. Clin J Am Soc Nephrol CJASN (2016) 11:1343–52. 10.2215/CJN.12051115 PMC4974890 27189318

[14] Vallon V Thomson SC. Renal Function in Diabetic Disease Models: The Tubular System in the Pathophysiology of the Diabetic Kidney. Annu Rev Physiol (2012) 74:351–75. 10.1146/annurev-physiol-020911-153333 PMC3807782 22335797

[15] Vallon V Thomson SC. The Tubular Hypothesis of Nephron Filtration and Diabetic Kidney Disease. Nat Rev Nephrol (2020) 16:317–36. 10.1038/s41581-020-0256-y PMC7242158 32152499

[16] DeFronzo RA Reeves WB. Pathophysiology of Diabetic Kidney Disease: Impact of SGLT2 Inhibitors. Nat Rev Nephrol (2021) 17:319–34. 10.1038/s41581-021-00393-8 33547417

[17] Thomson SC Vallon V. Effects of SGLT2 Inhibitor and Dietary NaCl on Glomerular Hemodynamics Assessed by Micropuncture in Diabetic Rats. Am J Physiol Renal Physiol (2021) 320:F761–71. 10.1152/ajprenal.00552.2020 PMC8174804 33645318

[18] Brenner BM. Hemodynamically Mediated Glomerular Injury and the Progressive Nature of Kidney Disease. Kidney Int (1983) 23:647–55. 10.1038/ki.1983.72 6336299

[19] Thomson SC Blantz RC. Glomerulotubular Balance, Tubuloglomerular Feedback, and Salt Homeostasis. J Am Soc Nephrol JASN (2008) 19:2272–5. 10.1681/ASN.2007121326 18322161

[20] Akhtar M Taha NM Nauman A Mujeeb IB Al-Nabet A. Diabetic Kidney Disease: Past and Present. Adv Anatomic Pathol (2020) 27:87–97. 10.1097/PAP.0000000000000257 31876542

[21] Yu SM Bonventre JV. Acute Kidney Injury and Progression of Diabetic Kidney Disease. Adv Chronic Kidney Dis (2018) 25:166–80. 10.1053/j.ackd.2017.12.005 PMC5898826 29580581

[22] Chen Y Lee K Ni Z He JC. Diabetic Kidney Disease: Challenges, Advances, and Opportunities. Kidney Dis (Basel Switzerland) (2020) 6:215–25. 10.1159/000506634 PMC7445658 32903946

[23] Tervaert TW Mooyaart AL Amann K Cohen AH Cook HT Drachenberg CB. Pathologic Classification of Diabetic Nephropathy. J Am Soc Nephrol JASN (2010) 21:556–63. 10.1681/ASN.2010010010 20167701

[24] An Y Xu F Le W Ge Y Zhou M Chen H. Renal Histologic Changes and the Outcome in Patients With Diabetic Nephropathy. Nephrol Dial Transplant (2015) 30:257–66. 10.1093/ndt/gfu250 25063425

[25] Mottl AK Gasim A Schober FP Hu Y Dunnon AK. Segmental Sclerosis and Extracapillary Hypercellularity Predict Diabetic Esrd. J Am Soc Nephrol JASN (2018) 29:694–703. 10.1681/ASN.2017020192 29180393 PMC5791055

[26] Fioretto P Mauer M Brocco E Velussi M Frigato F Muollo B. Patterns of Renal Injury in NIDDM Patients With Microalbuminuria. Diabetologia (1996) 39:1569–76. 10.1007/s001250050616 8960844

[27] Fioretto P Caramori ML Mauer M. The Kidney in Diabetes: Dynamic Pathways of Injury and Repair. The Camillo Golgi Lecture 2007. Diabetologia (2008) 51:1347–55. 10.1007/s00125-008-1051-7 18528679

[28] Di Vincenzo A Bettini S Russo L Mazzocut S Mauer M Fioretto P. Renal Structure in Type 2 Diabetes: Facts and Misconceptions. J Nephrol (2020) 33:901–7. 10.1007/s40620-020-00797-y PMC7557481 32656750

[29] Lane PH Steffes MW Fioretto P Mauer SM. Renal Interstitial Expansion in Insulin-Dependent Diabetes Mellitus. Kidney Int (1993) 43:661–7. 10.1038/ki.1993.95 8455365

[30] Kato M Natarajan R. Epigenetics and Epigenomics in Diabetic Kidney Disease and Metabolic Memory. Nat Rev Nephrol (2019) 15:327–45. 10.1038/s41581-019-0135-6 PMC6889804 30894700

[31] Jaikumkao K Pongchaidecha A Chatsudthipong V Chattipakorn SC Chattipakorn N Lungkaphin A. The Roles of Sodium-Glucose Cotransporter 2 Inhibitors in Preventing Kidney Injury in Diabetes. Biomed Pharmacother (2017) 94:176–87. 10.1016/j.biopha.2017.07.095 28759755

[32] Zha D Cheng H Li W Wu Y Li X Zhang L. High Glucose Instigates Tubulointerstitial Injury by Stimulating Hetero-Dimerization of Adiponectin and Angiotensin II Receptors. Biochem Biophys Res Commun (2017) 493:840–6. 10.1016/j.bbrc.2017.08.047 28870804

[33] Wei PZ Szeto CC. Mitochondrial Dysfunction in Diabetic Kidney Disease. Clin Chim Acta; Int J Clin Chem (2019) 496:108–16. 10.1016/j.cca.2019.07.005 31276635

[34] Pickering TG. Stress, Inflammation, and Hypertension. J Clin Hypertension (2007) 9:567–71. 10.1111/j.1524-6175.2007.06301.x PMC8109879 17617770

[35] Wei L Xiao Y Li L Xiong X Han Y Zhu X. The Susceptibility Genes in Diabetic Nephropathy. Kidney Dis (Basel Switzerland) (2018) 4:226–37. 10.1159/000492633 PMC6276750 30574499

[36] Jha JC Banal C Chow BS Cooper ME Jandeleit-Dahm K. Diabetes and Kidney Disease: Role of Oxidative Stress. Antioxid Redox Signaling (2016) 25:657–84. 10.1089/ars.2016.6664 PMC5069735 26906673

[37] Thallas-Bonke V Tan SM Lindblom RS Snelson M Granata C Jha JC. Targeted Deletion of NADPH-Oxidase Nox4 From Proximal Tubules is Dispensable for Diabetic Kidney Disease Development. Nephrol Dial Transplant (2020) 36:988–97. 10.1093/ndt/gfaa376 33367789

[38] Blantz RC. Phenotypic Characteristics of Diabetic Kidney Involvement. Kidney Int (2014) 86:7–9. 10.1038/ki.2013.552 24978373 PMC4076684

[39] Miyata T Suzuki N van Ypersele de Strihou C. Diabetic Nephropathy: Are There New and Potentially Promising Therapies Targeting Oxygen Biology? Kidney Int (2013) 84:693–702. 10.1038/ki.2013.74 23486514

[40] Jiang N Zhao H Han Y Li L Xiong S Zeng L. Hif-1α Ameliorates Tubular Injury in Diabetic Nephropathy Via HO-1-Mediated Control of Mitochondrial Dynamics. Cell Proliferation (2020) 53:e12909. 10.1111/cpr.12909 32975326 PMC7653251

[41] Zheng Z Zheng F. Immune Cells and Inflammation in Diabetic Nephropathy. J Diabetes Res (2016) 2016:1841690. 10.1155/2016/1841690 26824038 PMC4707326

[42] Lim AK Tesch GH. Inflammation in Diabetic Nephropathy. Mediators Inflammation (2012) 2012:146154. 10.1155/2012/146154 PMC3432398 22969168

[43] Mezzano S Aros C Droguett A Burgos ME Ardiles L Flores C. NF-Kappab Activation and Overexpression of Regulated Genes in Human Diabetic Nephropathy. Nephrol Dial Transplant (2004) 19:2505–12. 10.1093/ndt/gfh207 15280531

[44] Schmid H Boucherot A Yasuda Y Henger A Brunner B Eichinger F. Modular Activation of Nuclear Factor-Kappab Transcriptional Programs in Human Diabetic Nephropathy. Diabetes (2006) 55:2993–3003. 10.2337/db06-0477 17065335

[45] Lee FT Cao Z Long DM Panagiotopoulos S Jerums G Cooper ME. Interactions Between Angiotensin II and NF-KappaB-Dependent Pathways in Modulating Macrophage Infiltration in Experimental Diabetic Nephropathy. J Am Soc Nephrol JASN (2004) 15:2139–51. 10.1097/01.ASN.0000135055.61833.A8 15284299

[46] Kolati SR Kasala ER Bodduluru LN Mahareddy JR Uppulapu SK Gogoi R. Bay 11-7082 Ameliorates Diabetic Nephropathy by Attenuating Hyperglycemia-Mediated Oxidative Stress and Renal Inflammation Via NF-κb Pathway. Environ Toxicol Pharmacol (2015) 39:690–9. 10.1016/j.etap.2015.01.019 25704036

[47] Brenner BM Lawler EV Mackenzie HS. The Hyperfiltration Theory: A Paradigm Shift in Nephrology. Kidney Int (1996) 49:1774–7. 10.1038/ki.1996.265 8743495

[48] Tojo A Onozato ML Ha H Kurihara H Sakai T Goto A. Reduced Albumin Reabsorption in the Proximal Tubule of Early-Stage Diabetic Rats. Histochem Cell Biol (2001) 116:269–76. 10.1007/s004180100317 11685557

[49] Nielsen R Christensen EI Birn H. Megalin and Cubilin in Proximal Tubule Protein Reabsorption: From Experimental Models to Human Disease. Kidney Int (2016) 89:58–67. 10.1016/j.kint.2015.11.007 26759048

[50] De S Kuwahara S Saito A. The Endocytic Receptor Megalin and Its Associated Proteins in Proximal Tubule Epithelial Cells. Membranes (2014) 4:333–55. 10.3390/membranes4030333 PMC4194038 25019425

[51] Amsellem S Gburek J Hamard G Nielsen R Willnow TE Devuyst O. Cubilin Is Essential for Albumin Reabsorption in the Renal Proximal Tubule. J Am Soc Nephrol JASN (2010) 21:1859–67. 10.1681/ASN.2010050492 PMC3014001 20798259

[52] Zhuang Y Yasinta M Hu C Zhao M Ding G Bai M. Mitochondrial Dysfunction Confers Albumin-Induced NLRP3 Inflammasome Activation and Renal Tubular Injury. Am J Physiol Renal Physiol (2015) 308:F857–66. 10.1152/ajprenal.00203.2014 25694478

[53] Nishi Y Satoh M Nagasu H Kadoya H Ihoriya C Kidokoro K. Selective Estrogen Receptor Modulation Attenuates Proteinuria-Induced Renal Tubular Damage by Modulating Mitochondrial Oxidative Status. Kidney Int (2013) 83:662–73. 10.1038/ki.2012.475 23344476

[54] Takagaki Y Shi S Katoh M Kitada M Kanasaki K. Dipeptidyl Peptidase-4 Plays a Pathogenic Role in BSA-Induced Kidney Injury in Diabetic Mice. Sci Rep (2019) 9:7519. 10.1038/s41598-019-43730-5 31101909 PMC6525172

[55] van Timmeren MM Bakker SJ Vaidya VS Bailly V Schuurs TA Damman J. Tubular Kidney Injury Molecule-1 in Protein-Overload Nephropathy. Am J Physiol Renal Physiol (2006) 291:F456–64. 10.1152/ajprenal.00403.2005 16467126

[56] Otomo H Nara M Kato S Shimizu T Suganuma Y Sato T. Sodium-Glucose Cotransporter 2 Inhibition Attenuates Protein Overload in Renal Proximal Tubule Via Suppression of Megalin O-GlcNacylation in Progressive Diabetic Nephropathy. Metabol: Clin Exp (2020) 113:154405. 10.1016/j.metabol.2020.154405 33069809

[57] Chang J Yan J Li X Liu N Zheng R Zhong Y. Update on the Mechanisms of Tubular Cell Injury in Diabetic Kidney Disease. Front Med (2021) 8:661076. 10.3389/fmed.2021.661076 PMC8042139 33859992

[58] Tesch GH. Diabetic Nephropathy - Is This an Immune Disorder? Clin Sci (2017) 131:2183–99. 10.1042/CS20160636 28760771

[59] Verzola D Cappuccino L D’Amato E Villaggio B Gianiorio F Mij M. Enhanced Glomerular Toll-Like Receptor 4 Expression and Signaling in Patients With Type 2 Diabetic Nephropathy and Microalbuminuria. Kidney Int (2014) 86:1229–43. 10.1038/ki.2014.116 24786705

[60] Kelly KJ Dominguez JH. Rapid Progression of Diabetic Nephropathy is Linked to Inflammation and Episodes of Acute Renal Failure. Am J Nephrol (2010) 32:469–75. 10.1159/000320749 20956853

[61] Zheng JM Yao GH Cheng Z Wang R Liu ZH. Pathogenic Role of Mast Cells in the Development of Diabetic Nephropathy: A Study of Patients at Different Stages of the Disease. Diabetologia (2012) 55:801–11. 10.1007/s00125-011-2391-2 22130579

[62] Magri CJ Fava S. The Role of Tubular Injury in Diabetic Nephropathy. Eur J Internal Med (2009) 20:551–5. 10.1016/j.ejim.2008.12.012 19782912

[63] Singh DK Winocour P Farrington K. Mechanisms of Disease: The Hypoxic Tubular Hypothesis of Diabetic Nephropathy. Nat Clin Practice Nephrol (2008) 4:216–26. 10.1038/ncpneph0757 18268525

[64] Vallon V Komers R. Pathophysiology of the Diabetic Kidney. Compr Physiol (2011) 1:1175–232. 10.1002/cphy.c100049 PMC6029262 23733640

[65] Badrick T Turner P. The Uncertainty of the Egfr. Indian J Clin Biochem IJCB (2013) 28:242–7. 10.1007/s12291-012-0280-1 PMC3689324 24426218

[66] Porrini E Ruggenenti P Luis-Lima S Carrara F Jiménez A de Vries APJ. Time for a Critical Appraisal. Nat Rev Nephrol (2019) 15:177–90. 10.1038/s41581-018-0080-9 30518813

[67] Abbate M Zoja C Remuzzi G. How Does Proteinuria Cause Progressive Renal Damage? J Am Soc Nephrol JASN (2006) 17:2974–84. 10.1681/ASN.2006040377 17035611

[68] Yamanouchi M Furuichi K Hoshino J Ubara Y Wada T. Nonproteinuric Diabetic Kidney Disease. Clin Exp Nephrol (2020) 24:573–81. 10.1007/s10157-020-01881-0 PMC7271053 32236782

[69] Molitch ME Steffes M Sun W Rutledge B Cleary P de Boer IH. Development and Progression of Renal Insufficiency With and Without Albuminuria in Adults With Type 1 Diabetes in the Diabetes Control and Complications Trial and the Epidemiology of Diabetes Interventions and Complications Study. Diabetes Care (2010) 33:1536–43. 10.2337/dc09-1098 PMC2890355 20413518

[70] Porrini E Ruggenenti P Mogensen CE Barlovic DP Praga M Cruzado JM. Non-Proteinuric Pathways in Loss of Renal Function in Patients With Type 2 Diabetes. Lancet Diabetes Endocrinol (2015) 3:382–91. 10.1016/S2213-8587(15)00094-7 25943757

[71] Koye DN Magliano DJ Reid CM Jepson C Feldman HI Herman WH. Risk of Progression of Nonalbuminuric CKD to End-Stage Kidney Disease in People With Diabetes: The Cric (Chronic Renal Insufficiency Cohort) Study. Am J Kidney Dis (2018) 72:653–61. 10.1053/j.ajkd.2018.02.364 29784612

[72] Yamanouchi M Furuichi K Hoshino J. Nonproteinuric Versus Proteinuric Phenotypes in Diabetic Kidney Disease: A Propensity Score-Matched Analysis of a Nationwide, Biopsy-Based Cohort Study. Diabetes Care (2019) 42:891–902. 10.2337/dc18-1320 30833372

[73] Bhalla V Zhao B Azar KM Wang EJ Choi S Wong EC. Racial/Ethnic Differences in the Prevalence of Proteinuric and Nonproteinuric Diabetic Kidney Disease. Diabetes Care (2013) 36:1215–21. 10.2337/dc12-0951 PMC3631839 23238659

[74] Gan WZ Ramachandran V Lim CSY Koh RY. Omics-Based Biomarkers in the Diagnosis of Diabetes. J Basic Clin Physiol Pharmacol (2019) 31(2):20190120. 10.1515/jbcpp-2019-0120 31730525

[75] Van JA Scholey JW Konvalinka A. Insights Into Diabetic Kidney Disease Using Urinary Proteomics and Bioinformatics. J Am Soc Nephrol JASN (2017) 28:1050–61. 10.1681/ASN.2016091018 PMC5373465 28159781

[76] Cherney D Perkins BA Lytvyn Y Heerspink H Rodríguez-Ortiz ME Mischak H. The Effect of Sodium/Glucose Cotransporter 2 (SGLT2) Inhibition on the Urinary Proteome. PloS One (2017) 12:e0186910. 10.1371/journal.pone.0186910 29084249 PMC5662219

[77] Liu KD Yang W Go AS Anderson AH Feldman HI Fischer MJ. Urine Neutrophil Gelatinase-Associated Lipocalin and Risk of Cardiovascular Disease and Death in CKD: Results From the Chronic Renal Insufficiency Cohort (Cric) Study. Am J Kidney Dis (2015) 65:267–74. 10.1053/j.ajkd.2014.07.025 PMC4353671 25311702

[78] Smith ER Lee D Cai MM Tomlinson LA Ford ML McMahon LP. Urinary Neutrophil Gelatinase-Associated Lipocalin may Aid Prediction of Renal Decline in Patients With Non-Proteinuric Stages 3 and 4 Chronic Kidney Disease (CKD). Nephrol Dial Transplant (2013) 28:1569–79. 10.1093/ndt/gfs586 23328709

[79] Fu WJ Xiong SL Fang YG Wen S Chen ML Deng RT. Urinary Tubular Biomarkers in Short-Term Type 2 Diabetes Mellitus Patients: A Cross-Sectional Study. Endocrine (2012) 41:82–8. 10.1007/s12020-011-9509-7 21779943

[80] Assal HS Tawfeek S Rasheed EA El-Lebedy D Thabet EH. Serum Cystatin C and Tubular Urinary Enzymes as Biomarkers of Renal Dysfunction in Type 2 Diabetes Mellitus. Clin Med Insights Endocrinol Diabetes (2013) 6:7–13. 10.4137/CMED.S12633 23966807 PMC3738377

[81] Satirapoj B Aramsaowapak K Tangwonglert T Supasyndh O. Novel Tubular Biomarkers Predict Renal Progression in Type 2 Diabetes Mellitus: A Prospective Cohort Study. J Diabetes Res (2016) 2016:3102962. 10.1155/2016/3102962 27672664 PMC5031837

[82] Duan S Chen J Wu L Nie G Sun L Zhang C. Assessment of Urinary NGAL for Differential Diagnosis and Progression of Diabetic Kidney Disease. J Diabetes its Complications (2020) 34:107665. 10.1016/j.jdiacomp.2020.107665 32653382

[83] Yang YH He XJ Chen SR Wang L Li EM Xu LY. Changes of Serum and Urine Neutrophil Gelatinase-Associated Lipocalin in Type-2 Diabetic Patients With Nephropathy: One Year Observational Follow-Up Study. Endocrine (2009) 36:45–51. 10.1007/s12020-009-9187-x 19390997

[84] Siddiqui K Joy SS. Potential Role and Excretion Level of Urinary Transferrin, KIM-1, Rbp, MCP-1 and NGAL Markers in Diabetic Nephropathy. Diabetes Metab Syndrome Obes Targets Ther (2020) 13:5103–11. 10.2147/DMSO.S282166 PMC7780984 33408494

[85] Satirapoj B. Tubulointerstitial Biomarkers for Diabetic Nephropathy. J Diabetes Res (2018) 2018:2852398. 10.1155/2018/2852398 29577044 PMC5822931

[86] Han WK Bailly V Abichandani R Thadhani R Bonventre JV. Kidney Injury Molecule-1 (Kim-1): A Novel Biomarker for Human Renal Proximal Tubule Injury. Kidney Int (2002) 62:237–44. 10.1046/j.1523-1755.2002.00433.x 12081583

[87] van Timmeren MM van den Heuvel MC Bailly V Bakker SJ van Goor H Stegeman CA. Tubular Kidney Injury Molecule-1 (KIM-1) in Human Renal Disease. J Pathol (2007) 212:209–17. 10.1002/path.2175 17471468

[88] de Carvalho JA Tatsch E Hausen BS Bollick YS Moretto MB Duarte T. Urinary Kidney Injury Molecule-1 and Neutrophil Gelatinase-Associated Lipocalin as Indicators of Tubular Damage in Normoalbuminuric Patients With Type 2 Diabetes. Clin Biochem (2016) 49:232–6. 10.1016/j.clinbiochem.2015.10.016 26519090

[89] Panduru NM Sandholm N Forsblom C Saraheimo M Dahlström EH Thorn LM. Kidney Injury Molecule-1 and the Loss of Kidney Function in Diabetic Nephropathy: A Likely Causal Link in Patients With Type 1 Diabetes. Diabetes Care (2015) 38:1130–7. 10.2337/dc14-2330 25784666

[90] Colombo M Valo E McGurnaghan SJ Sandholm N Blackbourn LAK Dalton RN. Biomarker Panels Associated With Progression of Renal Disease in Type 1 Diabetes. Diabetologia (2019) 62:1616–27. 10.1007/s00125-019-4915-0 PMC6677704 31222504

[91] Nielsen SE Reinhard H Zdunek D Hess G Gutiérrez OM Wolf M. Tubular Markers Are Associated With Decline in Kidney Function in Proteinuric Type 2 Diabetic Patients. Diabetes Res Clin Pract (2012) 97:71–6. 10.1016/j.diabres.2012.02.007 22402306

[92] Satirapoj B Pooluea P Nata N Supasyndh O. Urinary Biomarkers of Tubular Injury to Predict Renal Progression and End Stage Renal Disease in Type 2 Diabetes Mellitus With Advanced Nephropathy: A Prospective Cohort Study. J Diabetes Complications (2019) 33:675–81. 10.1016/j.jdiacomp.2019.05.013 31227289

[93] Kammer M Heinzel A Willency JA Duffin KL Mayer G Simons K. Integrative Analysis of Prognostic Biomarkers Derived From Multiomics Panels Helps Discrimination of Chronic Kidney Disease Trajectories in People With Type 2 Diabetes. Kidney Int (2019) 96:1381–8. 10.1016/j.kint.2019.07.025 31679767

[94] Umapathy D Dornadula S Krishnamoorthy E Mariappanadar V Viswanathan V Ramkumar KM. Ykl-40: A Biomarker for Early Nephropathy in Type 2 Diabetic Patients and Its Association With Inflammatory Cytokines. Immunobiology (2018) 223:718–27. 10.1016/j.imbio.2018.07.020 30077474

[95] Schmidt IM Hall IE Kale S Lee S He CH Lee Y. Chitinase-Like Protein Brp-39/YKL-40 Modulates the Renal Response to Ischemic Injury and Predicts Delayed Allograft Function. J Am Soc Nephrol JASN (2013) 24:309–19. 10.1681/ASN.2012060579 PMC3559482 23291472

[96] Lee JH Kim SS Kim IJ Song SH Kim YK In Kim J. Clinical Implication of Plasma and Urine YKL-40, as a Proinflammatory Biomarker, on Early Stage of Nephropathy in Type 2 Diabetic Patients. J Diabetes Complications (2012) 26:308–12. 10.1016/j.jdiacomp.2012.04.012 22705282

[97] Rathcke CN Persson F Tarnow L Rossing P Vestergaard H. Ykl-40, a Marker of Inflammation and Endothelial Dysfunction, Is Elevated in Patients With Type 1 Diabetes and Increases With Levels of Albuminuria. Diabetes Care (2009) 32:323–8. 10.2337/dc08-1144 PMC2628702 18957531

[98] Al-Rubeaan K Siddiqui K. Assessment of the Diagnostic Value of Different Biomarkers in Relation to Various Stages of Diabetic Nephropathy in Type 2 Diabetic Patients. Sci Rep (2017) 7:2684. 10.1038/s41598-017-02421-9 28577020 PMC5457399

[99] Pena MJ Heinzel A Heinze G Alkhalaf A Bakker SJ Nguyen TQ. A Panel of Novel Biomarkers Representing Different Disease Pathways Improves Prediction of Renal Function Decline in Type 2 Diabetes. PloS One (2015) 10:e0120995. 10.1371/journal.pone.0120995 25973922 PMC4431870

[100] Banba N Nakamura T Matsumura M Kuroda H Hattori Y Kasai K. Possible Relationship of Monocyte Chemoattractant Protein-1 With Diabetic Nephropathy. Kidney Int (2000) 58:684–90. 10.1046/j.1523-1755.2000.00214.x 10916091

[101] Grandaliano G Gesualdo L Ranieri E Monno R Montinaro V Marra F. Monocyte Chemotactic Peptide-1 Expression in Acute and Chronic Human Nephritides: A Pathogenetic Role in Interstitial Monocytes Recruitment. J Am Soc Nephrol JASN (1996) 7:906–13. 10.1681/ASN.V76906 8793800

[102] Rovin BH Doe N Tan LC. Monocyte Chemoattractant Protein-1 Levels in Patients With Glomerular Disease. Am J Kidney Dis (1996) 27:640–6. 10.1016/S0272-6386(96)90097-9 8629622

[103] Morii T Fujita H Narita T Shimotomai T Fujishima H Yoshioka N. Association of Monocyte Chemoattractant Protein-1 With Renal Tubular Damage in Diabetic Nephropathy. J Diabetes its Complications (2003) 17:11–5. 10.1016/S1056-8727(02)00176-9 12505750

[104] Thrailkill KM Nimmo T Bunn RC Cockrell GE Moreau CS Mackintosh S. Microalbuminuria in Type 1 Diabetes Is Associated With Enhanced Excretion of the Endocytic Multiligand Receptors Megalin and Cubilin. Diabetes Care (2009) 32:1266–8. 10.2337/dc09-0112 PMC2699744 19366958

[105] Ma J Guan M Bowden DW Ng MC Hicks PJ Lea JP. Association Analysis of the Cubilin (CUBN) and Megalin (Lrp2) Genes With ESRD in African Americans. Clin J Am Soc Nephrol CJASN (2016) 11:1034–43. 10.2215/CJN.12971215 PMC4891762 27197912

[106] Bryniarski MA Yee BM Jaffri I Chaves LD Yu JA Guan X. Increased Megalin Expression in Early Type 2 Diabetes: Role of Insulin-Signaling Pathways. Am J Physiol Renal Physiol (2018) 315:F1191–207. 10.1152/ajprenal.00210.2018 29949391

[107] Mori KP Yokoi H Kasahara M Imamaki H Ishii A Kuwabara T. Increase of Total Nephron Albumin Filtration and Reabsorption in Diabetic Nephropathy. J Am Soc Nephrol JASN (2017) 28:278–89. 10.1681/ASN.2015101168 PMC5198272 27382987

[108] Christensen EI Kristoffersen IB Grann B Thomsen JS Andreasen A Nielsen R. A Well-Developed Endolysosomal System Reflects Protein Reabsorption in Segment 1 and 2 of Rat Proximal Tubules. Kidney Int (2020) 99:841–53. 10.1016/j.kint.2020.11.015 33340516

[109] Mori K Nakao K. Neutrophil Gelatinase-Associated Lipocalin as the Real-Time Indicator of Active Kidney Damage. Kidney Int (2007) 71:967–70. 10.1038/sj.ki.5002165 17342180

[110] Bangstad HJ Seljeflot I Berg TJ Hanssen KF. Renal Tubulointerstitial Expansion Is Associated With Endothelial Dysfunction and Inflammation in Type 1 Diabetes. Scandinavian J Clin Lab Invest (2009) 69:138–44. 10.1080/00365510802444080 18846477

[111] Malinda KM Ponce L Kleinman HK Shackelton LM Millis AJ. Gp38k, a Protein Synthesized by Vascular Smooth Muscle Cells, Stimulates Directional Migration of Human Umbilical Vein Endothelial Cells. Exp Cell Res (1999) 250:168–73. 10.1006/excr.1999.4511 10388530

[112] Segerer S Nelson PJ Schlöndorff D. Chemokines, Chemokine Receptors, and Renal Disease: From Basic Science to Pathophysiologic and Therapeutic Studies. J Am Soc Nephrol JASN (2000) 11:152–76. 10.1681/ASN.V111152 10616852

[113] Wada T Furuichi K Sakai N Iwata Y Yoshimoto K Shimizu M. Up-Regulation of Monocyte Chemoattractant Protein-1 in Tubulointerstitial Lesions of Human Diabetic Nephropathy. Kidney Int (2000) 58:1492–9. 10.1046/j.1523-1755.2000.00311.x 11012884

[114] Ibrahim S Rashed L. Correlation of Urinary Monocyte Chemo-Attractant Protein-1 With Other Parameters of Renal Injury in Type-II Diabetes Mellitus. Saudi J Kidney Dis Transplant (2008) 19:911–7. 18974575

[115] Christensen EI Birn H. Megalin and Cubilin: Multifunctional Endocytic Receptors. Nat Rev Mol Cell Biol (2002) 3:256–66. 10.1038/nrm778 11994745

[116] Terryn S Tanaka K Lengelé JP Olinger E Dubois-Laforgue D Garbay S. Tubular Proteinuria in Patients With HNF1α Mutations: HNF1α Drives Endocytosis in the Proximal Tubule. Kidney Int (2016) 89:1075–89. 10.1016/j.kint.2016.01.027 27083284

[117] Alicic RZ Cox EJ Neumiller JJ Tuttle KR. Incretin Drugs in Diabetic Kidney Disease: Biological Mechanisms and Clinical Evidence. Nat Rev Nephrol (2020) 17(4):227–44. 10.1038/s41581-020-00367-2 33219281

[118] Clark AJ Parikh SM. Targeting Energy Pathways in Kidney Disease: The Roles of Sirtuins, AMPK, and PGC1α. Kidney Int (2020) 99:828–40. 10.1016/j.kint.2020.09.037. PMC7987711 33307105

[119] Packer M. Role of Impaired Nutrient and Oxygen Deprivation Signaling and Deficient Autophagic Flux in Diabetic Ckd Development: Implications for Understanding the Effects of Sodium-Glucose Cotransporter 2-Inhibitors. J Am Soc Nephrol JASN (2020) 31:907–19. 10.1681/ASN.2020010010 PMC7217421 32276962

[120] Tonneijck L Muskiet MHA Blijdorp CJ Smits MM Twisk JW Kramer MHH. Renal Tubular Effects of Prolonged Therapy With the GLP-1 Receptor Agonist Lixisenatide in Patients With Type 2 Diabetes Mellitus. Am J Physiol Renal Physiol (2019) 316:F231–40. 10.1152/ajprenal.00432.2018 30353743

[121] Martins FL Bailey MA Girardi ACC. Endogenous Activation of Glucagon-Like Peptide-1 Receptor Contributes to Blood Pressure Control: Role of Proximal Tubule Na(+)/H(+) Exchanger Isoform 3, Renal Angiotensin II, and Insulin Sensitivity. Hypertension (2020) 76:839–48. 10.1161/HYPERTENSIONAHA.120.14868 32755467

[122] Scheen AJ. Pharmacokinetics of Dipeptidylpeptidase-4 Inhibitors. Diabetes Obes Metab (2010) 12:648–58. 10.1111/j.1463-1326.2010.01212.x 20590741

[123] Rosenstock J Perkovic V Johansen OE Cooper ME Kahn SE Marx N. Effect of Linagliptin vs Placebo on Major Cardiovascular Events in Adults With Type 2 Diabetes and High Cardiovascular and Renal Risk: The Carmelina Randomized Clinical Trial. Jama (2019) 321:69–79. 10.1001/jama.2018.18269 30418475 PMC6583576

[124] Groop PH Cooper ME Perkovic V Hocher B Kanasaki K Haneda M. Linagliptin and Its Effects on Hyperglycaemia and Albuminuria in Patients With Type 2 Diabetes and Renal Dysfunction: The Randomized MARLINA-T2D Trial. Diabetes Obes Metab (2017) 19:1610–9. 10.1111/dom.13041 PMC5655723 28636754

[125] Alicic RZ Neumiller JJ. Sodium-Glucose Cotransporter 2 Inhibition and Diabetic Kidney Disease. Diabetes (2019) 68:248–57. 10.2337/dbi18-0007 30665953

[126] Hou YC Zheng CM. Molecular Mechanisms of SGLT2 Inhibitor on Cardiorenal Protection. Int J Mol Sci 21 (2020) 21:7833. 10.3390/ijms21217833 PMC7660105 33105763

[127] Castañeda AM Dutra-Rufato A Juarez MJ Grosembacher L Gonzalez-Torres H Musso CG. Sodium-Glucose Cotransporter 2 Inhibitors (SGLT2i): Renal Implications. Int Urol Nephrology (2020) 53:291–9. 10.1007/s11255-020-02585-w 32767250

[128] Vallon V Schwark JR Richter K Hropot M. Role of Na(+)/H(+) Exchanger NHE3 in Nephron Function: Micropuncture Studies With S3226, an Inhibitor of NHE3. Am J Physiol Renal Physiol (2000) 278:F375–9. 10.1152/ajprenal.2000.278.3.F375 10710541

[129] Pessoa TD Campos LC Carraro-Lacroix L Girardi AC Malnic G. Functional Role of Glucose Metabolism, Osmotic Stress, and Sodium-Glucose Cotransporter Isoform-Mediated Transport on Na+/H+ Exchanger Isoform 3 Activity in the Renal Proximal Tubule. J Am Soc Nephrol JASN (2014) 25:2028–39. 10.1681/ASN.2013060588 PMC4147971 24652792

[130] Onishi A Fu Y Darshi M Crespo-Masip M Huang W Song P. Effect of Renal Tubule-Specific Knockdown of the Na(+)/H(+) Exchanger NHE3 in Akita Diabetic Mice. Am J Physiol Renal Physiol (2019) 317:F419–f434. 10.1152/ajprenal.00497.2018 31166707 PMC6732454

[131] Silva Dos Santos D Polidoro JZ Borges-Júnior FA Girardi ACC. Cardioprotection Conferred by Sodium-Glucose Cotransporter 2 Inhibitors: A Renal Proximal Tubule Perspective. Am J Physiol Cell Physiol (2020) 318:C328–c336. 10.1152/ajpcell.00275.2019 31721613

[132] Chung S Kim S Son M Kim M Koh ES Shin SJ. Empagliflozin Contributes to Polyuria Via Regulation of Sodium Transporters and Water Channels in Diabetic Rat Kidneys. Front Physiol (2019) 10:271. 10.3389/fphys.2019.00271 30941057 PMC6433843

[133] Onishi A Fu Y Patel R Darshi M Crespo-Masip M Huang W. A Role for Tubular Na(+)/H(+) Exchanger NHE3 in the Natriuretic Effect of the SGLT2 Inhibitor Empagliflozin. Am J Physiol Renal Physiol (2020) 319:F712–f728. 10.1152/ajprenal.00264.2020 32893663 PMC7642886

[134] Shin SJ Chung S Kim SJ Lee EM Yoo YH Kim JW. Effect of Sodium-Glucose Co-Transporter 2 Inhibitor, Dapagliflozin, on Renal Renin-Angiotensin System in an Animal Model of Type 2 Diabetes. PloS One (2016) 11:e0165703. 10.1371/journal.pone.0165703 27802313 PMC5089752

[135] Ansary TM Nakano D Nishiyama A. Diuretic Effects of Sodium Glucose Cotransporter 2 Inhibitors and Their Influence on the Renin-Angiotensin System. Int J Mol Sci (2019) 20:629. 10.3390/ijms20030629 PMC6387046 30717173

[136] Yoshimoto T Furuki T Kobori H Miyakawa M Imachi H Murao K. Effects of Sodium-Glucose Cotransporter 2 Inhibitors on Urinary Excretion of Intact and Total Angiotensinogen in Patients With Type 2 Diabetes. J Invest Med (2017) 65:1057–61. 10.1136/jim-2017-000445 PMC5812257 28596160

[137] Cherney DZ Perkins BA Soleymanlou N Maione M Lai V Lee A. Renal Hemodynamic Effect of Sodium-Glucose Cotransporter 2 Inhibition in Patients With Type 1 Diabetes Mellitus. Circulation (2014) 129:587–97. 10.1161/CIRCULATIONAHA.113.005081 24334175

[138] Cherney DZ Perkins BA Soleymanlou N Xiao F Zimpelmann J Woerle HJ. Sodium Glucose Cotransport-2 Inhibition and Intrarenal RAS Activity in People With Type 1 Diabetes. Kidney Int (2014) 86:1057–8. 10.1038/ki.2014.246 25360497

[139] Chen L LaRocque LM Efe O Wang J Sands JM Klein JD. Effect of Dapagliflozin Treatment on Fluid and Electrolyte Balance in Diabetic Rats. Am J Med Sci (2016) 352:517–23. 10.1016/j.amjms.2016.08.015 PMC5119919 27865300

[140] Masuda T Muto S Fukuda K Watanabe M Ohara K Koepsell H. Osmotic Diuresis by SGLT2 Inhibition Stimulates Vasopressin-Induced Water Reabsorption to Maintain Body Fluid Volume. Physiol Rep (2020) 8:e14360. 10.14814/phy2.14360 31994353 PMC6987478

[141] Nilsson LM Zhang L Bondar A Svensson D Wernerson A Brismar H. Prompt Apoptotic Response to High Glucose in SGLT-Expressing Renal Cells. Am J Physiol Renal Physiol (2019) 316:F1078–f1089. 10.1152/ajprenal.00615.2018 30864838 PMC6580252

[142] Vallon V Rose M Gerasimova M Satriano J Platt KA Koepsell H. Knockout of Na-glucose Transporter SGLT2 Attenuates Hyperglycemia and Glomerular Hyperfiltration But Not Kidney Growth or Injury in Diabetes Mellitus. Am J Physiol Renal Physiol (2013) 304:F156–67. 10.1152/ajprenal.00409.2012 PMC3543626 23152292

[143] Gembardt F Bartaun C Jarzebska N Mayoux E Todorov VT Hohenstein B. The SGLT2 Inhibitor Empagliflozin Ameliorates Early Features of Diabetic Nephropathy in BTBR Ob/Ob Type 2 Diabetic Mice With and Without Hypertension. Am J Physiol Renal Physiol (2014) 307:F317–25. 10.1152/ajprenal.00145.2014 24944269

[144] Oraby MA El-Yamany MF Safar MM Assaf N Ghoneim HA. Dapagliflozin Attenuates Early Markers of Diabetic Nephropathy in Fructose-Streptozotocin-Induced Diabetes in Rats. Biomed Pharmacother (2019) 109:910–20. 10.1016/j.biopha.2018.10.100 30551545

[145] Das NA Carpenter AJ Belenchia A Aroor AR Noda M Siebenlist U. Empagliflozin Reduces High Glucose-Induced Oxidative Stress and Mir-21-Dependent TRAF3IP2 Induction and RECK Suppression, and Inhibits Human Renal Proximal Tubular Epithelial Cell Migration and Epithelial-to-Mesenchymal Transition. Cell Signalling (2020) 68:109506. 10.1016/j.cellsig.2019.109506 31862399 PMC7493965

[146] Wang XX Levi J Luo Y Myakala K Herman-Edelstein M Qiu L. Sglt2 Protein Expression Is Increased in Human Diabetic Nephropathy: Sglt2 PROTEIN Inhibition DECREASES Renal LIPID Accumulation, INFLAMMATION, and THE Development OF Nephropathy IN Diabetic Mice. J Biol Chem (2017) 292:5335–48. 10.1074/jbc.M117.779520 PMC5392679 28196866

[147] Korbut AI Taskaeva IS Bgatova NP Muraleva NA Orlov NB Dashkin MV. Sglt2 Inhibitor Empagliflozin and DPP4 Inhibitor Linagliptin Reactivate Glomerular Autophagy in Db/Db Mice, a Model of Type 2 Diabetes. Int J Mol Sci (2020) 21:2987. 10.3390/ijms21082987 PMC7215949 32340263

[148] Han E Shin E Kim G Lee JY Lee YH Lee BW. Combining SGLT2 Inhibition With a Thiazolidinedione Additively Attenuate the Very Early Phase of Diabetic Nephropathy Progression in Type 2 Diabetes Mellitus. Front Endocrinol (2018) 9:412. 10.3389/fendo.2018.00412 PMC6060671 30072956

[149] Bonnet F Scheen AJ. Effects of SGLT2 Inhibitors on Systemic and Tissue Low-Grade Inflammation: The Potential Contribution to Diabetes Complications and Cardiovascular Disease. Diabetes Metab (2018) 44:457–64. 10.1016/j.diabet.2018.09.005 30266577

[150] Xu C Wang W Zhong J Lei F Xu N Zhang Y. Canagliflozin Exerts Anti-Inflammatory Effects by Inhibiting Intracellular Glucose Metabolism and Promoting Autophagy in Immune Cells. Biochem Pharmacol (2018) 152:45–59. 10.1016/j.bcp.2018.03.013 29551587

[151] O’Neill J Fasching A Pihl L Patinha D Franzén S Palm F. Acute SGLT Inhibition Normalizes O2 Tension in the Renal Cortex But Causes Hypoxia in the Renal Medulla in Anaesthetized Control and Diabetic Rats. Am J Physiol Renal Physiol (2015) 309:F227–34. 10.1152/ajprenal.00689.2014 26041448

[152] Sano M Takei M Shiraishi Y Suzuki Y. Increased Hematocrit During Sodium-Glucose Cotransporter 2 Inhibitor Therapy Indicates Recovery of Tubulointerstitial Function in Diabetic Kidneys. J Clin Med Res (2016) 8:844–7. 10.14740/jocmr2760w PMC5087622 27829948

[153] Sano M. Inter-Organ Communication Pathway Manifested by Non-physiological Stress to the Kidney in Type Ii Diabetic Patients -Why Are Diabetic Patients Prone to Develop Heart Failure? Internal Med (Tokyo Japan) (2020) 59:1–5. 10.2169/internalmedicine.2870-19 PMC6995696 31178515

[154] Lambers Heerspink HJ de Zeeuw D Wie L Leslie B List J. Dapagliflozin a Glucose-Regulating Drug With Diuretic Properties in Subjects With Type 2 Diabetes. Diabetes Obes Metab (2013) 15:853–62. 10.1111/dom.12127 PMC3906841 23668478

[155] Cai T Ke Q Fang Y Wen P Chen H Yuan Q. Sodium-Glucose Cotransporter 2 Inhibition Suppresses HIF-1α-Mediated Metabolic Switch From Lipid Oxidation to Glycolysis in Kidney Tubule Cells of Diabetic Mice. Cell Death Dis (2020) 11:390. 10.1038/s41419-020-2544-7 32444604 PMC7242894

[156] Shirakawa K Sano M. Sodium-Glucose Co-Transporter 2 Inhibitors Correct Metabolic Maladaptation of Proximal Tubular Epithelial Cells in High-Glucose Conditions. Int J Mol Sci (2020) 21:7676. 10.3390/ijms21207676 PMC7589591 33081406

[157] Tomita I Kume S Sugahara S Osawa N Yamahara K Yasuda-Yamahara M. Sglt2 Inhibition Mediates Protection From Diabetic Kidney Disease by Promoting Ketone Body-Induced mTORC1 Inhibition. Cell Metab (2020) 32:404–19.e6. 10.1016/j.cmet.2020.06.020 32726607

[158] Ndibalema AR Kabuye D Wen S. Empagliflozin Protects Against Proximal Renal Tubular Cell Injury Induced by High Glucose Via Regulation of Hypoxia-Inducible Factor 1-Alpha. Diabetes Metab Syndrome Obesity: Targets Ther (2020) 13:1953–67. 10.2147/DMSO.S243170 PMC7297363 32606855

[159] Wanner C Inzucchi SE Lachin JM Fitchett D von Eynatten M Mattheus M. Empagliflozin and Progression of Kidney Disease in Type 2 Diabetes. New Engl J Med (2016) 375:323–34. 10.1056/NEJMoa1515920 27299675

[160] Neal B Perkovic V Mahaffey KW de Zeeuw D Fulcher G Erondu N. Canagliflozin and Cardiovascular and Renal Events in Type 2 Diabetes. New Engl J Med (2017) 377:644–57. 10.1056/NEJMoa1611925 28605608

[161] Perkovic V de Zeeuw D Mahaffey KW Fulcher G Erondu N Shaw W. Canagliflozin and Renal Outcomes in Type 2 Diabetes: Results From the CANVAS Program Randomised Clinical Trials. Lancet Diabetes Endocrinol (2018) 6:691–704. 10.1016/S2213-8587(18)30141-4 29937267

[162] Perkovic V Jardine MJ Neal B Bompoint S Heerspink HJL Charytan DM. Canagliflozin and Renal Outcomes in Type 2 Diabetes and Nephropathy. New Engl J Med (2019) 380:2295–306. 10.1056/NEJMoa1811744 30990260

[163] Wiviott SD Raz I Bonaca MP Mosenzon O Kato ET Cahn A. Dapagliflozin and Cardiovascular Outcomes in Type 2 Diabetes. New Engl J Med (2019) 380:347–57. 10.1056/NEJMoa1812389 30415602

[164] Heerspink HJL Stefánsson BV Correa-Rotter R Chertow GM Greene T Hou FF. Dapagliflozin in Patients With Chronic Kidney Disease. New Engl J Med (2020) 383:1436–46. 10.1056/NEJMoa2024816 32970396

[165] Jensen J Omar M Kistorp C Tuxen C Gustafsson I Køber L. Effects of Empagliflozin on Estimated Extracellular Volume, Estimated Plasma Volume, and Measured Glomerular Filtration Rate in Patients With Heart Failure (Empire HF Renal): A Prespecified Substudy of a Double-Blind, Randomised, Placebo-Controlled Trial. Lancet Diabetes Endocrinol (2020) 9:106–16. 10.1016/S2213-8587(20)30382-X 33357505

[166] Cannon CP Pratley R Dagogo-Jack S Mancuso J Huyck S Masiukiewicz U. Cardiovascular Outcomes With Ertugliflozin in Type 2 Diabetes. N Engl J Med (2020) 383:1425–35. 10.1056/NEJMoa2004967 32966714

[167] Sarafidis P Ortiz A Ferro CJ Halimi JM Kreutz R Mallamaci F. Sodium–Glucose Co-Transporter-2 Inhibitors for Patients With Diabetic and Nondiabetic Chronic Kidney Disease: A New Era has Already Begun. J Hypertension (2021) 39:1090–7. 10.1097/HJH.0000000000002776 33443971

[168] Wanner C. Empa-Reg OUTCOME: The Nephrologist’s Point of View. Am J Cardiol (2017) 120:S59–67. 10.1016/j.amjcard.2017.05.012 28606346

[169] Sarafidis P Ferro CJ Morales E Ortiz A Malyszko J Hojs R. SGLT-2 Inhibitors and GLP-1 Receptor Agonists for Nephroprotection and Cardioprotection in Patients With Diabetes Mellitus and Chronic Kidney Disease. A Consensus Statement by the EURECA-m and the DIABESITY Working Groups of the ERA-EDTA. Nephrol Dial Transplant (2019) 34:208–30. 10.1093/ndt/gfy407 30753708

[170] Herrington WG Preiss D Haynes R von Eynatten M Staplin N Hauske SJ. The Potential for Improving Cardio-Renal Outcomes by Sodium-Glucose Co-Transporter-2 Inhibition in People With Chronic Kidney Disease: A Rationale for the EMPA-KIDNEY Study. Clin Kidney J (2018) 11:749–61. 10.1093/ckj/sfy090 PMC6275453 30524708

[171] Davies MJ D’Alessio DA Fradkin J Kernan WN Mathieu C. Management of Hyperglycemia in Type 2 Diabetes, 2018. A Consensus Report by the American Diabetes Association (ADA) and the European Association for the Study of Diabetes (Easd). Diabetes Care (2018) 41:2669–701. 10.2337/dci18-0033 PMC6245208 30291106

[172] Cosentino F Grant PJ Aboyans V Bailey CJ Ceriello A Delgado V. 2019 ESC Guidelines on Diabetes, Pre-Diabetes, and Cardiovascular Diseases Developed in Collaboration With the EASD. Eur Heart J (2020) 41:255–323. 10.1093/eurheartj/ehz486 31497854

[173] Buse JB Wexler DJ. 2019 Update to: Management of Hyperglycaemia in Type 2 Diabetes, 2018. A Consensus Report by the American Diabetes Association (ADA) and the European Association for the Study of Diabetes (Easd). Diabetologia (2020) 63:221–8. 10.1007/s00125-019-05039-w 31853556

[174] Marx N Davies MJ Grant PJ Mathieu C Petrie JR Cosentino F. Guideline Recommendations and the Positioning of Newer Drugs in Type 2 Diabetes Care. Lancet Diabetes Endocrinol (2021) 9:46–52. 10.1016/S2213-8587(20)30343-0 33159841 PMC12140926

[175] Kidney Disease: Improving Global Outcomes (KDIGO) Diabetes Work Group Kdigo 2020 Clinical Practice Guideline for Diabetes Management in Chronic Kidney Disease. Kidney Int (2020) 98:S1–s115. 10.1016/j.kint.2020.06.019 32998798

[176] Kleinaki Z Kapnisi S Theodorelou-Charitou SA Nikas IP Paschou SA. Type 2 Diabetes Mellitus Management in Patients With Chronic Kidney Disease: An Update. Hormones (2020) 19:467–76. 10.1007/s42000-020-00212-y 32500461

